# Single extracellular vesicle detection assay identifies membrane-associated α-synuclein as an early-stage biomarker in Parkinson’s disease

**DOI:** 10.1016/j.xcrm.2025.101999

**Published:** 2025-03-07

**Authors:** Shijun Yan, Wenjing Zhang, Xinying Li, Suman Dutta, Andrew R. Castle, Yiming Liu, Anis Sahoo, Chor Lai Lam, Nicholas J.F. Gatford, Michele T. Hu, Chen-zhong Li, Cheng Jiang, Bowen Shu, George K. Tofaris

**Affiliations:** 1 Nuffield Department of Clinical Neurosciences & Kavli Institute for Nanoscience Discovery, University of Oxford, Oxford, UK; 2 School of Medicine, The Chinese University of Hong Kong, Shenzhen, People's Republic of China; 3 Dermatology Hospital, Southern Medical University, Guangzhou, People's Republic of China

**Keywords:** droplet microfluidics, neuronally derived extracellular vesicles, biomarker, immunoassay, Parkinson’s disease diagnostics, prodromal, RBD, L1CAM, aggregation, α-synuclein

## Abstract

Accurate diagnosis of early Parkinson’s disease requires platforms suitable for detecting minute amounts of neuronally derived biomarkers in the massive protein excess of easily accessible biofluids such as blood. Here, we describe an on-chip droplet-confined fluorescence reporting assay that identified α-synuclein on the membrane of L1CAM+ extracellular vesicles (EVs) immunocaptured from human serum and corroborate this finding by super-resolution direct stochastic optical reconstruction microscopy (dSTORM) microscopy. Using conditioned media from neuroblastoma cells expressing α-synuclein mutants or patient-derived induced pluripotent stem cell (iPSC) neurons with α-synuclein gene triplication, we found that association of α-synuclein with the L1CAM+ EV surface is increased under pathological conditions. Accordingly, this readout, as measured by the droplet-based assay, is an improved predictive biomarker in the prodromal phase (area under the receiver operating characteristic curve [AUC] = 0.93) or diagnostic biomarker in the clinical phase (AUC = 0.95) of Parkinson’s disease. More broadly, our platform will simplify the assessment of EV membrane proteins and facilitate their application as diagnostic biomarkers across diverse clinical indications.

## Highlights

• Droplet-based assay for precise multiplexed quantification of EV membrane proteins
• α-Synuclein is associated with the membrane on the surface of L1CAM+ neuronal EVs
• α-Synuclein L1CAM+ EV membrane association increases under pathological conditions
• EV membrane α-synuclein is an improved biomarker for early Parkinson’s diagnostics

## Graphical abstract

**Figure:**
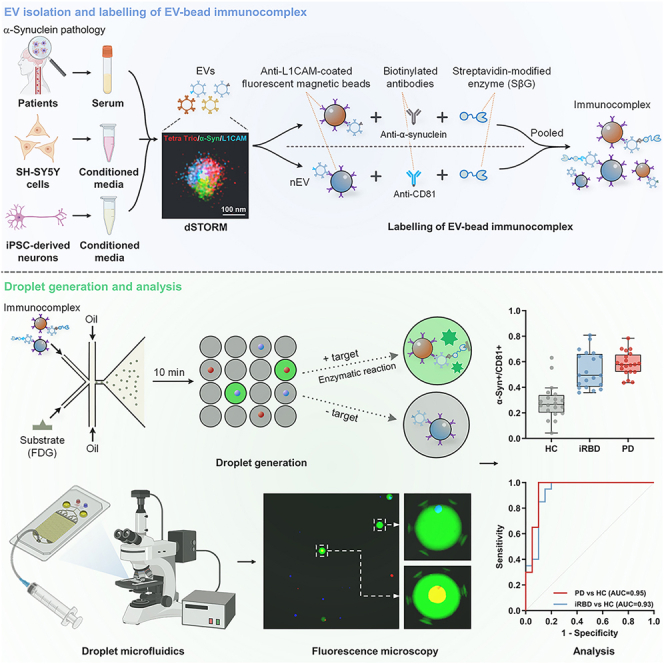

## Teaser

Yan et al. develop a droplet-based microfluidic immunoassay for multiplexed quantification of membrane-associated proteins at single-extracellular-vesicle (EV) resolution. They identify membrane-associated α-synuclein on the surface of neuronal EVs and demonstrate that it is increased under pathological conditions and in individuals at risk of or with Parkinson’s disease.

## Introduction

Parkinson’s disease (PD) is the most common movement disorder with a prolonged prodromal phase during which a number of non-motor symptoms already manifest. Pathologically, PD is causatively linked to the intraneuronal aggregation of α-synuclein. Isolated rapid eye movement (REM) sleep behavior disorder (iRBD) is the strongest predictor of α-synucleinopathy with >80% of subjects converting primarily to PD or dementia with Lewy bodies (DLB) within 12 years^1^ Currently, PD diagnosis relies on the clinical assessment of the cardinal motor signs, posing challenges for early-stage detection and differentiation from PD-like conditions. There is no established blood test capable of identifying the underlying pathology. Molecular pathology based on liquid biopsy has transformed the diagnosis of other indications, but the detection of rare neuronal markers in accessible but complex biofluids such as blood remains challenging. Measurement of total or pathological forms of free α-synuclein in serum or plasma revealed high heterogeneity across studies and overall poor performance.^2^

Extracellular vesicles (EVs) are membranous nanoparticles that are secreted by all cell types including neurons and reach the systemic circulation. For this reason, they have emerged as a rich source of biomarkers due to their capacity to transport tissue- or disease-specific molecular signals.^3^ Isolation of presumed neuronally derived L1CAM+ EVs (L1EVs), which mirror the homeostatic state of nerve cells, has shown promise as a biomarker for neurodegenerative disorders.^4^^,^^5^^,^^6^ Our group previously developed a clean immunocapture assay for L1EV isolation from serum and demonstrated that α-synuclein in bulk L1EV lysates as determined by Meso Scale Discovery (MSD) electrochemiluminescence assay is increased in individuals at risk of or with PD and related dementia.^7^^,^^8^^,^^9^ However, EV subpopulations are often heterogeneous, and the detection of relevant proteins requires large sample input (typically >200 μL) and sophisticated instruments that may not always be easily accessible. In this respect, the development of next-generation platforms, such as droplet microfluidic immunoassays,^10^^,^^11^^,^^12^^,^^13^ offer an ultrasensitive alternative technology to conventional bulk analysis of EV-enriched samples using minimal sample volume (<20 μL) for single EV protein analysis.^14^^,^^15^

In this study, we introduce an improved ultrasensitive platform for EV membrane-associated protein detection and demonstrate its application in PD diagnostics. This technique involves the immunocapture of serum L1EVs followed by on-chip droplet compartmentalization of individual EV-bead complexes, forming a monolayer droplet array for enzyme-catalyzed fluorescence imaging. Harnessing the superior resolution of this platform, we found that α-synuclein is detectable on the membrane of L1EVs isolated from human serum. Using conditioned media derived from neuroblastoma cells expressing α-synuclein mutants or human induced pluripotent stem cell (iPSC)-derived neurons from a patient with α-synuclein gene triplication (SNCA^TRIP^), we demonstrate that this association is increased under pathological conditions, is detectable by antibodies that recognize aggregated forms, and is partly determined by the membrane-binding affinity of PD-associated mutations. Accordingly, we showed that, when corrected to the generic EV transmembrane protein CD81, L1EV membrane-associated α-synuclein differentiates individuals with iRBD or PD from healthy controls (HCs) with high accuracy (area under the receiver operating characteristic [ROC] curve [AUC] > 0.93). Thus, L1EV membrane-associated α-synuclein offers an improved predictive and diagnostic blood test for neuronal α-synucleinopathy.

## Results

### Development of the microfluidic digital immunoassay for single EV detection

**Figure 1:**
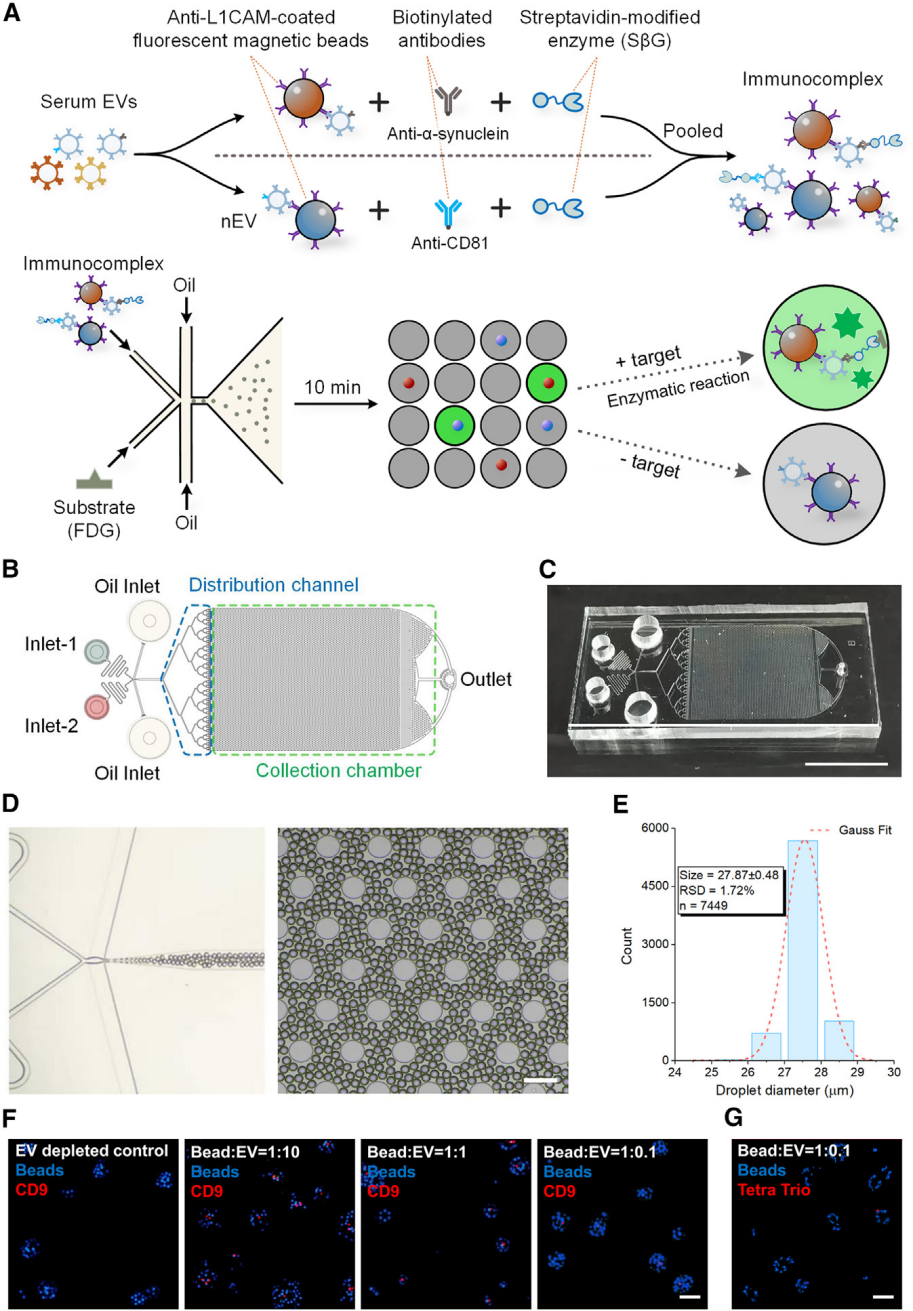
Droplet-based microfluidic extracellular vesicle digital immunoassay (A) Workflow of the assay, also demonstrated in Video S1. (B) Schematic illustration of the droplet microfluidic device with further design details in Figure S1. (C) Photograph of the microfluidic device (75 × 25 × 1.1 mm); scale bar, 2 cm. (D) Representative images of the picoliter-sized droplets; scale bar, 100 μm. (E) Size distribution of the droplets averaging 27.9 μm in diameter with further analysis in Figures S2–S4. (F) Representative fluorescence microscopy image of L1EVs immunocaptured from serum using DAPI-labeled beads, followed by staining with Alexa Fluor 647-labeled anti-CD9 in EV-depleted serum as a control and bead-to-serum-EV ratio of 1:10, or 1:1, or 1:0.1; scale bar, 2 μm. (G) Representative fluorescence microscopy image of L1EVs immunocaptured from serum using DAPI-labeled beads, followed by staining with Alexa Fluor 561-labeled anti-Tetraspanin Trio (CD9, CD63, and CD81) using a bead-to-serum-EV ratio of 1:0.1; scale bar, 2 μm.

The microfluidic chip (Figure S1) generated picoliter-scale droplets containing a single immunocaptured EV, which is labeled with biotinylated antibodies against the target surface protein, conjugated to streptavidin-β-galactosidase (SβG) and visualized with fluorescein substrate (fluorescein-di-β-D-galactopyranoside, FDG) as summarized in Figures 1A–1C. The chip facilitated sample mixing and droplet generation through the differential pressure driven by withdrawing the piston of a connecting syringe. This generated approximately 200,000 monodisperse picoliter-sized droplets with an average diameter of 27.9 μm in a high-throughput format (Figures 1D and 1E), while consuming minimal sample at a piston withdrawal volume of 1 mL (Figure S2). In this way, approximately 10% of droplets contained a fluorescently encoded magnetic bead with or without a single EV immunocomplex. In the absence of an EV or the target EV membrane-associated protein, droplet-confined beads display solely the coded bead fluorescence rather than green fluorescence. Therefore, the design enables the visualization of a unique enzyme-labeled immunocomplex per bead targeting an EV membrane antigen that is amplified by the enzymatic reporter SβG (Video S1). The assay sensitivity is determined by the ratio of green fluorescent droplets to the total “bead only”-containing droplet (red or blue) count, referred to as % of EV+ beads. This readout can be converted to % of EVs expressing the target per mL of biofluid using the Poisson distribution.

Video S1. Assay workflow, related to Figure 1

To confirm that the majority of droplets encapsulated no more than one bead, we performed serial dilutions of input beads (Figure S3A). We found that, at a concentration of 1.4 × 10^7^ beads/mL, 9.8% of the droplets contained a single bead, while 90.1% of the droplets did not contain a bead, which is consistent with the Poisson distribution prediction (Figures S3B and S3C). To optimize the reaction time for enzyme-mediated fluorescence at this optimal concentration, we observed the time course of fluorescent signal generation. The enzymatic fluorescence peaked at 10 min, while no positive droplet was found when no substrate was added as a control (Figure S4).

To maximize the probability of capturing a single EV per bead per droplet, we introduced a 10-fold excess of beads relative to the number of EVs as determined by nanoparticle tracking analysis (NTA). To confirm that a single EV was captured per bead, we used fluorescent microscopy on serially diluted serum that was subjected to immunocapture with anti-L1CAM antibodies followed by EV visualization with Alexa 647-labeled anti-CD9 antibodies. We found that multiple EVs were captured per bead at 1:10 or 1:1 (bead: EV) ratio, whereas, at 1:0.1 (bead: EV) ratio, a single L1EV was captured per bead (Figure 1F). The suitability of 1:0.1 (bead: EV) ratio was further confirmed with Tetraspanin Trio (Tetra Trio) antibody cocktail (against CD9, CD63, and CD81), which is more likely to visualize all EVs (Figure 1G). Thus, under these conditions, >99.9% of the bead-positive droplets contained only one target EV, thereby enabling accurate single-EV membrane protein measurement. In control experiments, we tested and confirmed that no signal was detected in EV-depleted serum (Figure 1F) and demonstrated successful EV immunocapture from serum with either anti-CD9- or anti-L1CAM-coated beads by immunoblotting, NTA, and negative staining transmission electron microscopy (TEM) as summarized in Figure S5.

### The assay enables parallel quantification of distinct EV subpopulations

**Figure 2:**
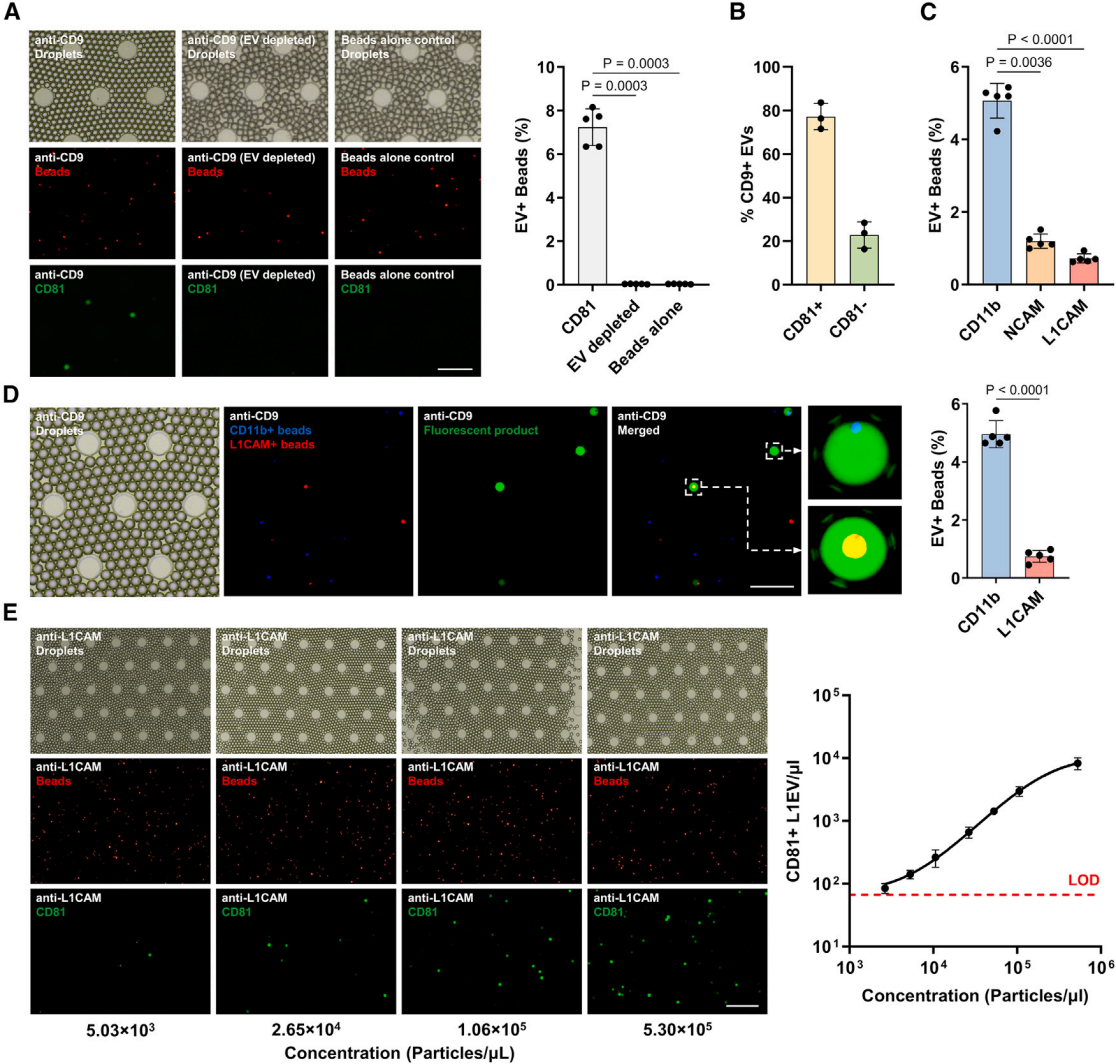
Singleplex and multiplex EV membrane protein detection (A) Representative images and quantification of CD81 on CD9 immunocaptured EVs from human serum, EV-depleted serum, and beads alone control using Cy5-labeled beads, as measured by the droplet assay; *n* = 5 individuals with PD tested; scale bar, 150 μm. Confirmation of serum EV immunocapture is shown in Figure S5. (B) Histogram showing the membrane-associated CD81 expression on single CD9+ EVs isolated from serum, as measured by nano flow cytometry; *n* = 3 individuals with PD tested; also see Figure S6. (C) Detection of CD11b, NCAM, and L1CAM on CD9 immunocaptured EVs from serum using the droplet assay; *n* = 5 individuals with PD tested. (D) Representative images and quantification of duplex CD11b (DAPI-labeled beads) and L1CAM (Cy5-labeled beads) on CD9 immunocaptured EVs from serum, as measured by the droplet assay; *n* = 5 individuals with PD tested; scale bar, 100 μm. (E) Representative images and calibration curve of CD81 detection on L1EVs using the droplet assay at various EV concentrations as measured by NTA; *n* = 3 independent experiments. Scale bar, 250 μm. Data are represented as mean ± SD. Statistical significance was determined by one-way ANOVA (A and C) or student’s two-tailed t test (D).

Having optimized the conditions for single EV detection, we performed direct serum EV immunocapture using Cy5-labeled beads conjugated to antibodies against the generic marker CD9, followed by on-chip detection of surface proteins that are either generic markers (e.g., CD81) or denote specific EV subpopulations (e.g., CD11b, NCAM, and L1CAM). Quantification throughout was performed by calculating the ratio of green fluorescent droplets (denoting a target EV-bead immunocomplex) to red fluorescent beads (denoting the total number of beads) and is referred to as % of EV+ beads as stated earlier. In control experiments, we included EV-depleted serum following ultracentrifugation or serum incubated with beads alone not conjugated to antibodies. These experiments confirmed that the signal generated by CD81 immunoreactivity arises specifically from CD9+ EVs, as no signal was seen in controls (Figure 2A). Quantification of the signal (Figure 2A) revealed that CD81 was detected in 7.2% EV+ beads, corresponding to 80.0% of serum CD9+ EVs as estimated by Poisson distribution, in agreement with our previous measurements for these generic EV markers using immunoblotting.^16^

To further validate the accuracy of the droplet immunoassay, we used nano flow cytometry (NanoFCM) to analyze membrane-associated CD9 and CD81 expression on single serum EVs isolated using ultracentrifugation. This analysis revealed that CD81 was detected in 77.2% of serum CD9+ EVs (Figures 2B and S6), which aligns with the result obtained with our platform. In control experiments, we tested and confirmed that negligible signal was detected in the antibodies-only control without sample condition (Figure S6).

We then evaluated the performance of the platform in measuring the abundance of EV subpopulations using antibodies against neuronal (L1CAM or NCAM) or immune (CD11b) markers after serum immunocapture with an antibody against CD9 which is a generic EV membrane marker. We found that the immune cell marker CD11b was detected in 5.3% EV+ beads, and the neuronal markers NCAM or L1CAM were detected in 1.2% and 0.7% EV+ beads, respectively (Figure 2C). These estimates correspond to 58.3% of CD9+ serum EVs for CD11b+ EVs, 13.2% for NCAM+ EVs, and 8.0% for L1EVs.

To assess the multiplexing capability of the platform, we performed direct anti-CD9 immunocapture from serum using either DAPI-labeled or Cy5-labeled beads, followed by incubation with SβG-biotinylated detection antibodies against either CD11b (for DAPI-labeled beads) or L1CAM (Cy5-labeled beads), respectively. The enzyme-labeled EV-bead immunocomplexes were then mixed and loaded with FDG onto the platform for quantification. In this setup, we found that CD11b was detected in 5.0% EV+ beads and L1CAM was detected in 0.7% EV+ beads (Figure 2D). These estimates correspond to 54.8% of CD9+ serum EVs for CD11b+ EVs and 8.2% for L1EVs, which concur with our singleplex measurements. These data show that our platform can be reliably used for the simultaneous multiplex assessment of target proteins on different EV subpopulations.

To further validate the efficacy of our platform in measuring signal arising from a single EV of low abundance, we focused subsequent experiments on L1EVs. We performed serial dilutions of serum followed by immunocapture with anti-L1CAM and quantified CD81 immunofluorescence. CD81+L1EVs exhibited excellent correlation with NTA results (Figure 2E). We calculated the limit of detection (LOD) as 3 standard deviations above the background and the limit of quantification (LOQ) as 10 standard deviations above the background for our assay. Based on these calculations, the LOD is 66 CD81+ L1EVs/μL, the LOQ is 112 CD81+ L1EVs/μL, and the dynamic range is 66 to 8,307 CD81+ L1EVs/μL.

### Identification of α-synuclein as a serum EV membrane-associated protein

**Figure 3:**
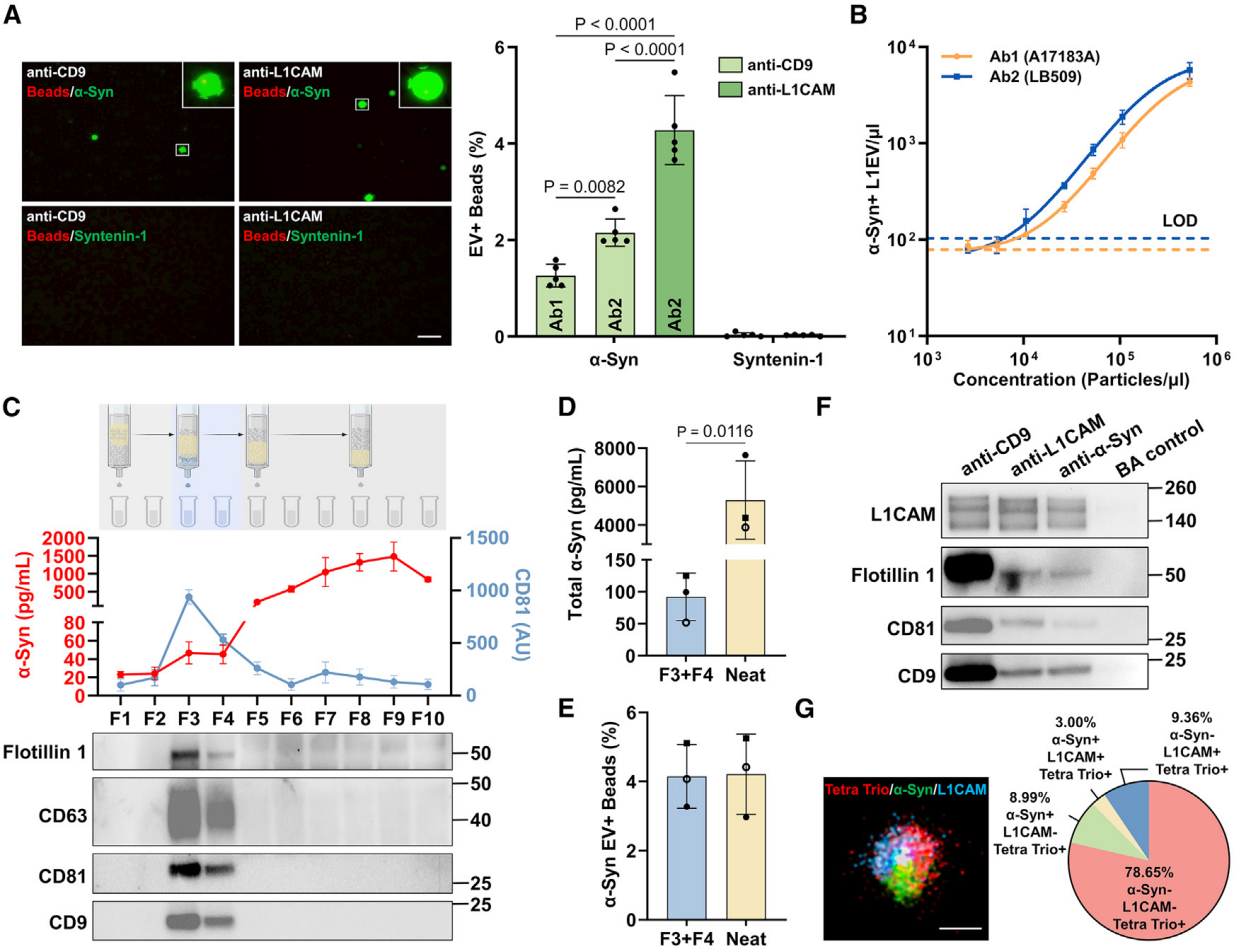
Identification of α-synuclein as a serum EV membrane-associated protein (A) Representative images and quantification of membrane-associated α-synuclein (α-Syn) and the internal EV marker syntenin-1 on CD9+ EVs or L1EVs isolated from serum using the droplet assay. Anti-α-Syn antibody clone A17183A, which recognizes mainly the aggregated forms of α-Syn under native conditions (Ab1), or clone LB509, which recognizes a C-terminal region spanning amino acid 115–125 of total α-Syn (Ab2), was used; *n* = 5 individuals with PD tested; scale bar, 100 μm. (B) Assay calibration of L1EV membrane-associated α-Syn using Ab1 or Ab2 at various EV concentrations as measured by NTA; *n* = 3 individuals with PD tested. LOD and LOQ values are summarized in Table S1. (C) SEC illustration, MSD electrochemiluminescence detection of α-Syn and the EV marker CD81, and immunoblotting of 10 eluted fractions (F) from 1 mL of serum. (D and E) (D) MSD electrochemiluminescence detection of α-Syn in neat serum and pooled SEC F3+F4 and (E) detection of α-Syn on L1EVs immunocaptured from neat serum or pooled SEC F3+F4 using the droplet assay; also see Figure S7; *n* = 3 individuals with PD tested. (F) Immunocapture using anti-CD9, anti-L1CAM, and anti-α-Syn from F3+F4 followed by immunoblotting, with beads alone (BA) used as a control. (G) Representative image of a single EV obtained with dSTORM and corresponding quantification showing the % colocalization of L1CAM and α-Syn on Tetraspanin Trio+ (Tetra Trio+) EVs from SEC F3+F4; pooled serum from 5 individuals with PD was tested; scale bar, 100 nm. Data are represented as mean ± SD. Statistical significance was determined by one-way ANOVA (A) or student’s two-tailed t test (D).

Since α-synuclein binds to lipid membranes either physiologically via its amphipathic helical conformation or pathologically when aggregated,^17^ we asked whether it is detectable on the EV membrane. To this end, we mixed anti-CD9 or anti-L1CAM immunocaptured EVs from PD patient serum with FDG substrate and two different SβG-labeled-biotinylated anti-α-synuclein antibodies: antibody clone A17183A, which recognizes mainly the aggregated form of α-synuclein (Ab1),^18^ or antibody clone LB509, which recognizes an epitope in the C-terminal region spanning amino acid 115–125 of α-synuclein (Ab2).^19^ Interestingly, potentially aggregated α-synuclein was detected in 1.3% CD9+ EV+ beads using Ab1, corresponding to 14.0% of CD9+ EVs (Figure 3A). Total α-synuclein was detected on 4.3% of L1CAM+ EV+ beads and 2.2% of CD9+ EV+ beads using Ab2, corresponding to 47.3% of L1EVs and 23.8% of CD9+ EVs, respectively. In control experiments, we tested and confirmed that, under the same conditions, the internal EV protein syntenin-1 showed negligible signal (Figure 3A). These data indicate that physiological or aggregated forms of α-synuclein are associated with the membrane on the EV surface, and these conformers are enriched on L1EVs.

To assess the performance of our droplet-based assay for detecting α-synuclein immunoreactive EVs, we quantified α-synuclein immunofluorescence in L1EVs isolated from serially diluted serum with either Ab1 (A17183A) or Ab2 (LB509) as shown in Figure 3B. This analysis for Ab1 and Ab2, respectively, established an LOD of 79 or 103 α-synuclein+ L1EVs/μL, an LOQ of 131 or 176 α-synuclein+ L1EVs/μL, and a dynamic range from 79 to 4,328 or 103 to 5,759 α-synuclein+ L1EVs/μL (Table S1). The calculated minimum volume for the detection of L1EV membrane-associated α-synuclein is approximately 3 μL of serum. These are the estimated limits of the assay, and further validation in patient samples was performed with 20 μL of serum as the starting volume.

To further validate this finding and exclude non-specific association of free α-synuclein, we separated the EVs from the bulk of free human serum proteins, which includes free α-synuclein, using size exclusion chromatography (SEC). As we described previously,^20^ EVs were primarily eluted and enriched in fraction (F) 3 and F4, as determined by MSD electrochemiluminescence detection of the EV marker CD81 and immunoblotting for the surface EV markers CD63, CD81, CD9, and the internal EV cargo protein Flotillin 1 (Figure 3C). In contrast, most α-synuclein in the serum was found in a free form in later fractions as measured by MSD electrochemiluminescence (Figure 3C). In neat serum, total α-synuclein as quantified by MSD was 5,294.1 ± 1,666.9 pg per mL whereas total α-synuclein in SEC F3+F4 was 92.0 ± 37.2 pg per mL, indicating that SEC F3+F4 are indeed depleted of excess free α-synuclein (Figure 3D). We then performed anti-L1CAM immunocapture from either neat serum or EV-enriched SEC F3+F4 followed by duplex droplet-based measurements of total α-synuclein using LB509 antibody or CD9 (*n* = 3). These experiments showed that α-synuclein and CD9 on L1EVs as detected by our platform were similar in neat serum and SEC F3+F4, i.e., irrespective of the great excess of free α-synuclein in the former (Figures 3E, S7A, and S7B). Thus, free α-synuclein in the serum does not associate non-specifically with L1EVs during the isolation process and does not interfere with the specificity of the microfluidic assay. To further assess whether α-synuclein is on the EV surface, we performed immunocapture with anti-CD9, anti-L1CAM, or anti-α-synuclein (using antibody clone Syn1) from SEC F3+F4 followed by immunoblotting. These experiments confirmed that the generic EV markers CD9, CD81, and Flotillin 1 were detected following anti-α-synuclein immunocapture whereas no bands were observed in the negative control (Figure 3F). Direct detection of α-synuclein by immunoblotting is not possible as its abundance in EVs is below the LOD of this method.

We confirmed these findings further using direct stochastic optical reconstruction microscopy (dSTORM) to visualize the colocalization of tetraspanins CD9, CD63, CD81 (Tetra Trio), L1CAM, and α-synuclein (using antibody clone MJFR1) on serum EVs isolated by SEC. These experiments identified individual EVs co-stained with fluorescently labeled antibodies against Tetra Trio (Alexa Fluor 561), L1CAM (Alexa Fluor 488), and α-synuclein (Alexa Fluor 647) as shown in Figure 3G. Quantification revealed that α-synuclein was detected on the surface of 11.99% of total EVs (8.99% α-Syn+/L1CAM-/Tetra Trio+ and 3.00% α-Syn+/L1CAM+/Tetra Trio+) and 24.27% of L1EVs (the ratio of 3.00% α-Syn+/L1CAM+/Tetra Trio+ to 12.36% L1CAM+/Tetra Trio+) as shown in Figure 3G. Thus, membrane-associated α-synuclein is found on a subpopulation of serum EVs, and it is enriched in L1EVs, which are thought to arise from neurons.

### α-synuclein L1EV membrane association increases under pathological conditions

**Figure 4:**
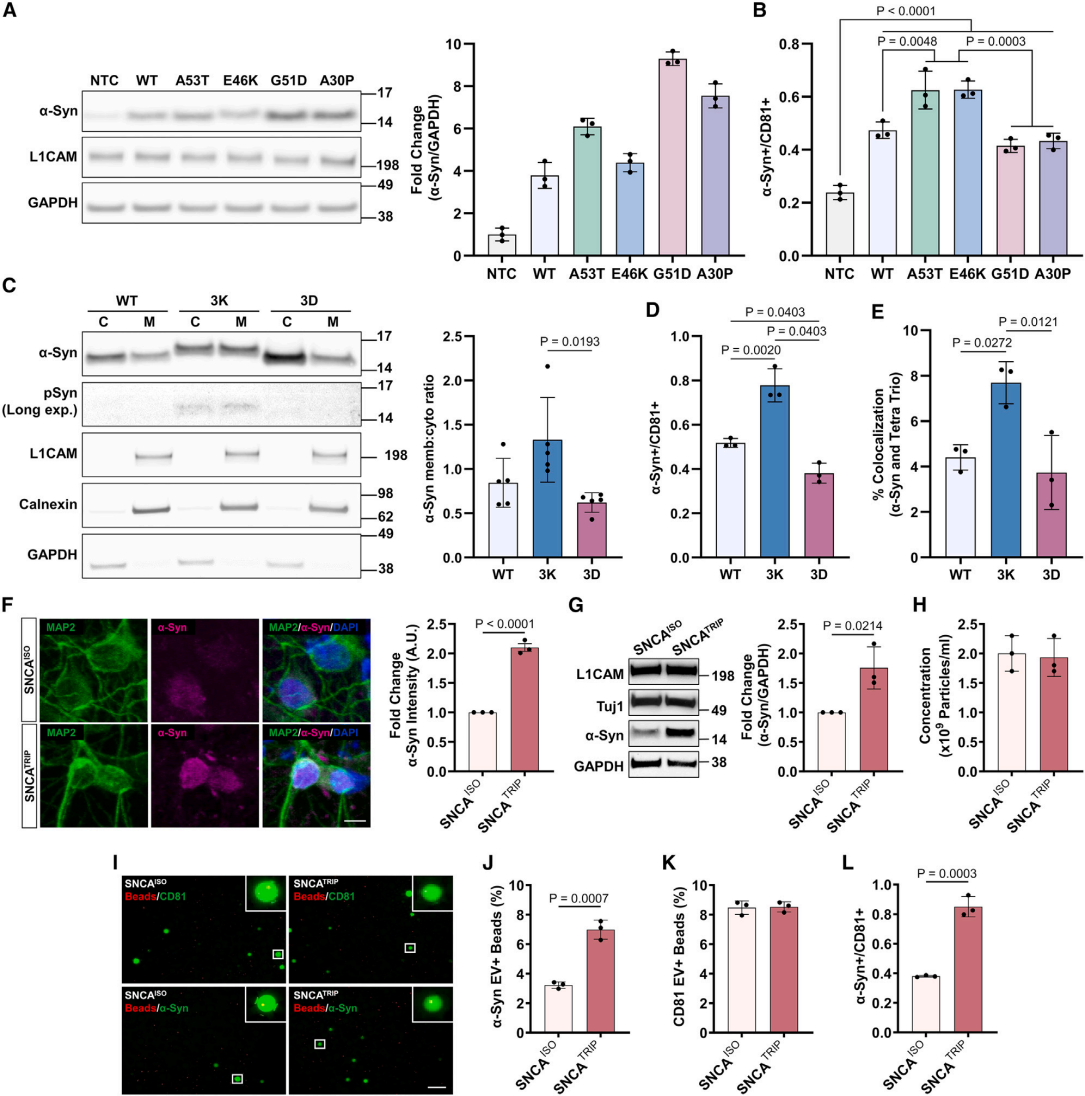
L1EV membrane-associated α-synuclein is increased under pathological conditions (A) Immunoblotting of RIPA-soluble fraction from SH-SY5Y cells expressing PD-associated α-synuclein (α-Syn) mutations A53T, E46K, G51D, and A30P. (B) A53T and E46K mutant α-Syn increased the L1EV membrane-associated α-Syn/CD81 ratio compared to non-transduced control (NTC), wild-type (WT), G51D, and A30P as measured by the droplet assay. Confirmation of CM L1EV immunocapture is shown in Figure S8. (C) Expression of 3K mutant α-Syn in SH-SY5Y cells increased the membrane (memb)-to-cytosol (cyto) ratio compared to the expression of the 3D mutant α-Syn; *n* = 5 independent experiments. (D) 3K mutant α-Syn increased the L1EV membrane association and α-Syn/CD81 ratio compared to WT and 3D, whereas expression of 3D mutant α-Syn decreased L1EV membrane association as measured by the droplet assay. (E) Quantification by dSTORM of membrane-associated α-Syn on individual EVs using antibody clone 5G4, which recognizes mainly the aggregated forms of α-Syn under native conditions. (F) Confocal images and corresponding quantification of iPSC-derived SNCA^ISO^ and SNCA^TRIP^ dopaminergic neurons stained with MAP2 and α-Syn, showing an approximately 2-fold increase in α-Syn immunofluorescence in the SNCA^TRIP^ line; scale bar, 5 μm. (G) Immunoblotting showed an approximately 2-fold increase in endogenous α-Syn expression when normalized to GAPDH in SNCA^TRIP^ neuronal lysates comparted to isogenic controls. (H) NTA of EVs in the neuronal CM showed that the total EV concentration is similar between the two lines and is not affected by pathological α-Syn expression. (I) Representative images of membrane-associated α-Syn and CD81 detection on L1EVs from neuronal CM as measured by the droplet assay; scale bar, 100 μm. (J) Membrane-associated α-Syn was increased in L1EVs isolated from SNCA^TRIP^ neurons compared to SNCA^ISO^ control L1EVs as measured by the droplet assay. (K) No difference in membrane-associated CD81 in L1EVs from SNCA^TRIP^ neurons compared to SNCA^ISO^ control L1EVs as measured by the droplet assay, in agreement with the NTA results. (L) L1EV membrane-associated α-Syn/CD81 ratio was increased by approximately 2-fold in SNCA^TRIP^ when compared to SNCA^ISO^ control L1EVs as measured by the droplet assay. *n* = 3 independent experiments (SH-SY5Y cells) unless stated otherwise or *n* = 3 independent differentiations (iPSC-derived neurons). Data are represented as mean ± SD. Statistical significance was determined by Kruskal-Wallis test (C), one-way ANOVA (B, D, and E), or student’s two-tailed t test (F–J and L). See also Figure S9 for further analysis.

To investigate whether L1EV surface α-synuclein is a pathological association, we expressed either wild-type (WT) or PD-associated mutant α-synuclein (A53T, E46K, G51D, and A30P) in SH-SY5Y cells, which endogenously express L1CAM (Figure 4A). The culture media were conditioned for 48 h, and successful EV immunocapture with anti-L1CAM-coated beads from CM was confirmed as shown in Figure S8. The cells were lysed in RIPA buffer and fractionated with ultracentrifugation into RIPA-soluble and RIPA-insoluble fractions. WT and mutant α-synuclein were detected in the RIPA-soluble fraction as shown in Figure 4A, but not in the RIPA-insoluble fraction (Figure S9A). We then performed L1EV immunocapture followed by on-chip duplex detection of total α-synuclein (LB509 antibody) and CD81 (Figures S9B and S9C). Under these conditions, we found that intracellular α-synuclein levels are a critical determinant of L1EV membrane association (when compared to endogenous expression) based on EV α-synuclein/CD81 ratio as shown in Figure 4B. In addition, we observed that the E46K mutation, which increases lipid binding,^21^ further increased L1EV membrane association when expressed equally to the WT protein, whereas the G51D and A30P mutations, which are known to decrease lipid binding,^22^^,^^23^ were associated with L1EV membrane similar to WT protein (Figure 4B), even though their intracellular expression was higher than the WT protein (Figure 4A). These data suggest that L1EV membrane association is, at least partly, also determined by the effect of the pathogenic mutations on lipid binding.

To further corroborate this observation, we generated α-synuclein mutants that were previously shown to exacerbate the lipid binding affinity of single-point mutations. Specifically, the engineered mutant 3K (E35K + E46K + E61K) enhances whereas the engineered mutant 3D (V40D + G51D + V66D) reduces the membrane association of their corresponding single PD-associated mutations (E46K vs. G51D).^24^ α-Synuclein WT, 3K, and 3D were expressed in SH-SY5Y cells, and the media were conditioned for 48 h and collected for L1EV isolation, while the cells were subjected to subcellular fractionation into digitonin-soluble (cytosol) and Triton X-100-soluble (membrane) fractions according to a previously published α-synuclein fractionation protocol.^25^ Immunoblotting confirmed that expression of 3K mutant α-synuclein increased the membrane (memb)-to-cytosol (cyto) ratio compared to WT, whereas expression of 3D mutant α-synuclein decreased the memb/cyto ratio (Figure 4C). L1EV immunocapture from corresponding CM was followed by on-chip duplex detection of total α-synuclein (LB509 antibody) or CD81 (Figures S9D and S9E). We found that expression of the 3K mutant that binds more avidly to membranes inside the cells also led to increased L1EV membrane association as quantified by the α-synuclein/CD81 ratio relative to WT or 3D (Figure 4D). It is noteworthy that, under these conditions, 3K expression was associated with weak phosphorylation at Ser129 (Figure 4C), suggesting that this L1EV membrane association reflects a pathological form of α-synuclein, as demonstrated previously inside the cells. This was further corroborated by dSTORM using Tetra Trio to visualize all EVs and the 5G4 antibody, which recognizes primarily aggregated α-synuclein under native conditions.^18^ In this experiment, EVs were isolated from CM using differential centrifugation followed by filtration and ultracentrifugation. We observed that the percentage of Tetra Trio+ (Alexa Fluor 561) and α-synuclein+ (Alexa Fluor 647) EVs was increased in 3K- compared to 3D-expressing cells (Figure 4E).

To confirm that membrane-associated α-synuclein is present on EVs released by human neurons and is increased under pathological conditions detectable by the droplet-based assay, we isolated L1EVs from CM taken from patient-derived iPSC dopaminergic neuronal cultures. An iPSC line from a patient with α-synuclein gene (*SNCA*) triplication (SNCA^TRIP^), which is associated with higher α-synuclein expression and more severe disease, and the isogenic iPSC clone (SNCA^ISO^) were differentiated into dopaminergic neurons (Table S2). We confirmed an approximately 2-fold increase in intracellular α-synuclein levels in SNCA^TRIP^ compared to SNCA^ISO^ neuronal cultures^26^ as evident from α-synuclein immunostaining (Figure 4F) and immunoblotting (Figure 4G), without any significant difference in the neuronal markers L1CAM and Tuj1 across the two lines (Figure 4G). Culture media conditioned from day 52 to day 60 were similarly enriched in EVs as measured by NTA (Figure 4H). L1EV immunocapture from CM was then performed, followed by on-chip detection of either total α-synuclein using LB509 antibody or CD81 (Figure 4I). We found that membrane-associated α-synuclein was increased in L1EVs from SNCA^TRIP^ neurons compared to SNCA^ISO^ control L1EVs (Figure 4J). In agreement with our NTA measurements, CD81 quantification was similar across the two conditions (Figure 4K). α-Synuclein/CD81 ratio also differentiated SNCA^TRIP^ from SNCA^ISO^ neuronal cultures (Figure 4L). To further corroborate these findings, we used SEC followed by MSD electrochemiluminescence to quantify α-synuclein and CD81 levels in iPSC-derived EV-enriched fractions. This experiment further confirmed an increase in the α-synuclein/CD81 ratio in EVs from SNCA^TRIP^ neurons compared to its isogenic control (Figure S9F). Thus, L1EVs released from human neurons with SNCA^TRIP^ exhibit a 2-fold increase in EV membrane-associated α-synuclein levels when compared to SNCA^ISO^ L1EVs, which reflects the increased α-synuclein expression in SNCA^TRIP^ neurons.^26^ Collectively, these data show that α-synuclein is found at least in part on the surface of EVs and this phenomenon is increased with pathogenic protein mutations or overexpression.

### Serum L1EV membrane-associated α-synuclein is increased in individuals with iRBD or PD compared to HCs

**Figure 5:**
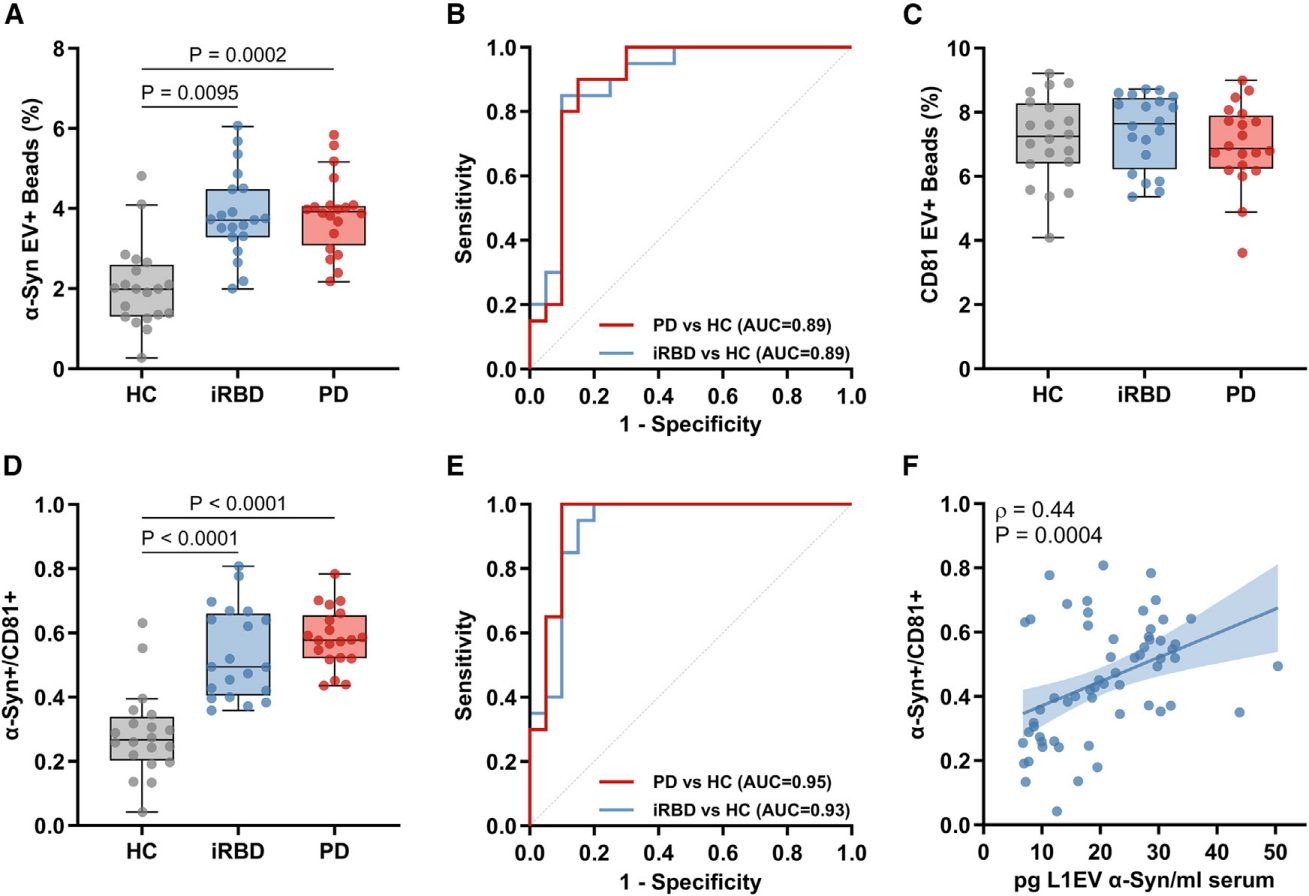
Serum L1EV membrane-associated-α-synuclein is increased in individuals with iRBD or PD (A and B) (A) Boxplot and (B) corresponding receiver operating characteristic (ROC) curves of L1EV membrane-associated α-synuclein (α-Syn) measurements using the LB509 antibody for healthy controls (HCs) and individuals with iRBD or PD. (C) Boxplot of L1EV membrane-associated CD81 measurements for HCs and individuals with iRBD or PD. (D and E) (D) Boxplot and (E) corresponding ROC curves of L1EV membrane-associated α-Syn/CD81 ratio for HCs and individuals with iRBD or PD. (F) Correlation between total α-Syn levels in serum L1EV lysates as measured by MSD electrochemiluminescence and L1EV membrane-associated α-Syn/CD81 ratio as measured by the droplet-based immunoassay. *n* = 20 individuals per group. The midline of the boxplots indicates the median, and the box indicates the 25th and 75th percentiles (A, C, and D). Statistical significance was determined by Kruskal-Wallis test (A, C, and D). Least-squares regression line with 95% confidence interval (CI) was plotted, and Spearman coefficient was reported (F). Data on assay reproducibility are shown in Figure S10.

To investigate the clinical utility of our platform and the potential of L1EV membrane-associated α-synuclein as a biomarker in patients, we assessed serum samples from age- and gender-matched individuals with the diagnosis of iRBD (*n* = 20) or PD (*n* = 20) and HCs (*n* = 20, total *n* = 60) from the Oxford Discovery cohort as summarized in Table 1. All sample processing and analyses were performed blinded to the diagnosis. We found that patients with iRBD or PD exhibited increased L1EV membrane-associated α-synuclein compared to HCs as shown in Figure 5A. Individuals with iRBD were differentiated from controls with an AUC of 0.89, as shown in Figure 5B. Similarly, patients with sporadic PD were differentiated from controls with an AUC of 0.89. There was no significant difference in CD81 quantification across these groups (Figure 5C). We also assessed the α-synuclein/CD81 ratio, which adjusts for the number of EVs detected across groups. Interestingly, L1EV α-synuclein/CD81 ratio exhibited an improved performance in differentiating individuals with either iRBD or PD from controls compared to the measurements of α-synuclein alone (Figure 5D). Specifically, α-synuclein/CD81 ratio differentiated individuals with iRBD from controls with an AUC of 0.93 and PD patients from controls with an AUC of 0.95 (Figure 5E).

**Table 1:** Demographic characteristics

	HC	iRBD	Sporadic PD	*p* value
No of individuals	20	20	20	N/A
Gender, no. (%)	–			
Female	5 (25.0)	3 (15.0)	5 (25.0)	0.68
Male	15 (75.0)	17 (85.0)	15 (75.0)	
Age, mean (SD), y	65.28 (12.53)	66.35 (6.15)	66.49 (8.39)	0.91
MoCA, mean (SD)	27.05 (2.04)	26.50 (2.88)	25.68 (2.56)	0.17
L1EV α-Syn, median (IQR), pg/mL^a^	10.03 (5.96)	19.78 (12.78)	28.36 (8.21)	<0.0001
α-Syn EV+ beads, median (IQR), %^b^	1.98 (1.16)	3.72 (1.29)	3.85 (0.75)	<0.0001
CD81 EV+ beads, median (IQR), %^b^	7.50 (1.55)	7.65 (1.85)	6.87 (1.41)	0.64
α-Syn+/CD81+, median (IQR), %^b^	0.26 (0.11)	0.49 (0.24)	0.56 (0.09)	<0.0001

Data are number (%), mean (SD), or median (IQR).

Abbreviations: iRBD, isolated REM sleep behavior disorder; PD, Parkinson’s disease; MoCA, Montreal Cognitive Assessment; L1EV, L1CAM positive extracellular vesicles; α-Syn, α-synuclein.

One-way ANOVA was used to compare age and MoCA; Kruskal-Wallis one-way analysis of variance was used to compare L1EV α-Syn, α-Syn EV+ beads, CD81 EV+ beads, and α-Syn+/CD81+ ratio; and Pearson χ^2^ test was used to compare gender frequency between groups.

a Measurements of total α-Syn in L1EV using MSD electrochemiluminescence.

b Quantification of L1EV membrane-associated α-Syn and CD81 using the droplet assay.

To further evaluate the clinical potential of L1EV membrane α-synuclein as a biomarker, we assessed the reproducibility of the assay by examining the same sample in triplicates or two separate samples from the same patient (*n* = 3 individuals). We estimated the inter-assay coefficient of variation to be 4.45% (Figure S10A). We also found that L1EV membrane-associated α-synuclein as measured by the droplet-based assay (Figures S10B–S10D) or total L1EV α-synuclein levels as measured by MSD electrochemiluminescence (Figure S10E) were consistent across two samples from the same patient despite variability in total/free serum α-synuclein as determined by MSD electrochemiluminescence (Figure S10F). We thus asked whether single-EV measurements of membrane-associated α-synuclein correlate with our previous quantification of total α-synuclein in bulk lysates of L1EVs using MSD electrochemiluminescence.^7^^,^^8^ This analysis revealed a correlation (ρ = 0.44, *p* = 0.0004) between L1EV membrane-associated α-synuclein/CD81 ratio and L1EV total α-synuclein levels (Figure 5F).

## Discussion

We describe an improved analytical platform designed for the ultrasensitive detection and quantification of EV membrane-associated proteins at single-EV resolution. By compartmentalizing individual EVs captured on magnetic beads into tens of thousands of microdroplets, the assay enables the detection of low-abundance EV subtypes such as neuronally derived EVs, with an estimated LOD of 66 CD81+ L1EVs/μL, surpassing the detection limit of previously reported nanoplasmonic^27^ or nanosensor-based^28^ EV detection techniques and standard ELISA measurements by several orders of magnitude. Our assay has a number of advantages compared to previously reported droplet-based EV prototypes applied in cancer^10^ such as speed (approximately 10 min) and simplicity as it can be implemented by a syringe-vacuum-driven microfluidic droplet chip with a standard fluorescence microscope, while maintaining analytical performance comparable to those performed by exclusive equipment such as a fluorescence spectrophotometer.

Importantly, unlike other microfluidic EV detection platforms,^10^^,^^11^ our assay enables multiplexing of diverse EV membrane-associated proteins at single-EV resolution from an estimated minimum volume of ∼3 μL of serum, which is well within the blood volume (∼35 μL) required for “pin-prick” tests. These features are highly desirable for potential clinical use in point-of-care settings. Thus, our technology offers a more detailed representation of EV heterogeneity and a more comprehensive understanding of individual EV changes from minimal sample volumes. This is especially useful for the detection of rare neuronally relevant EV biomarkers that are diluted in the massive excess of serum proteins. In this respect, an important discovery enabled by the assay is the identification of membrane-associated α-synuclein as a biomarker that is detected already in individuals with iRBD, i.e., it precedes the diagnosis of PD. Interestingly, membrane-associated α-synuclein was detected in the minority of the total (CD9+) EV population in PD and was especially enriched in neuronally derived L1EVs based on two complementary methods and confirmed in neuronal culture media. This finding suggests that the membrane-associated fraction of α-synuclein and the capability to measure it by our platform offer a more sensitive assay of neuronal homeostasis. Accordingly, we found that L1EV membrane-associated α-synuclein is increased under pathological conditions in cell culture and exhibited an improved performance (AUC = 0.95 for PD) when compared to our previously reported (AUC = 0.86 for PD) bulk measurements in lysed EVs.^8^

In solution, α-synuclein adopts an α-helical conformation in its N-terminal domain in the presence of membranes with acidic phospholipids or high curvature^17^ as in the case of EVs,^29^ but binding to membranous vesicles isolated from human samples has not been systematically evaluated in patients or demonstrated on EVs. The interaction of α-synuclein with membranes was shown to promote the formation of physiological multimers^30^ or, under certain conditions, to induce aggregation.^31^^,^^32^^,^^33^ *In vitro*, membrane-associated α-synuclein acquires a “pathological” conformation depending on the lipid composition and the α-synuclein-to-lipid ratio,^34^ but to what extent such changes occur in patient-derived EVs requires further investigation. Therefore, it is presently unknown how much of the signal we detected in patients reflects increased mislocalization of physiological α-synuclein on the EV surface or its assembly into a pathological conformation with differential antibody binding affinity. The assay utilized the LB509 antibody (BioLegend), which binds to amino acids 115–125 of α-synuclein^19^ and has a higher affinity for monomeric than fibrillar α-synuclein.^35^ α-Synuclein was also detectable on EVs with antibodies (clone A17183A and 5G4) that reportedly recognize primarily oligomeric/aggregated forms of α-synuclein under native conditions and at concentrations higher than those used in our assay.^18^ The latter suggests that a fraction of α-synuclein on the L1EV membrane exhibits a pathological assembly or conformation, and refinement of the assay using conformation-specific antibodies could further enhance the sensitivity and specificity of the biomarker.

Our results in cells expressing PD-associated α-synuclein mutations or in iPSC-derived dopaminergic neuronal cultures with SNCA^TRIP^ suggest that the association of α-synuclein with EV membranes reflects, at least partly, a pathological state. What regulates the binding of α-synuclein on the EV membrane is currently unclear and requires further investigation. Our cell-based experiments show that intracellular α-synuclein levels and the lipid binding affinity of α-synuclein, as determined by disease-associated and engineered mutations, are two critical determinants of incorporation into the EV membrane. We propose that α-synuclein on the EV membrane could signify the formation of a conformer of α-synuclein that may accumulate in close vicinity to membranous organelles during EV biogenesis or is induced by changes in their lipid composition. Our comparative analysis of L1EV membrane-associated α-synuclein in neat and SEC-processed serum suggests that a potential protein corona that has been reported in some EV preparations^36^ or free α-synuclein does not compound the reproducibility of the result when measurements are performed at single-EV resolution.

Although further validation is required in larger cohorts, our results suggest that L1EV membrane-associated α-synuclein may offer an improved predictive biomarker for PD compared to bulk measurements in EV lysates.^7^^,^^8^ Alongside our previous work,^7^^,^^8^^,^^9^ these findings reinforce the notion that L1EVs isolated from serum exhibit altered composition in neurodegenerative diseases well before the onset of disabling symptoms, thus offering a promising means of developing clinical screening tests that are pertinent to specific brain pathologies. In this regard, the capability afforded by our platform for precise quantification of EV subpopulations signifies a substantial progress toward this goal.

### Limitations of the study

The sensitivity demonstrated in our experimental setup does not fully reflect the inherent potential of this detection method, primarily due to throughput restrictions. The speed limitations in acquiring and processing fluorescent images resulted in the analysis of only approximately 10% of the input beads, which compromises the assay sensitivity. Future improvements could integrate automated video-based image acquisition and quantification^37^ or on-chip EV immunocapture, such as the one we have recently described.^38^ A device incorporating such elements could facilitate rapid EV biomarker detection in a form not dissimilar to current “pin-prick” tests for other indications and clinical use in point-of-care settings.

## Resource availability

### Lead contact

Further information and request for resources and reagents should be directed to and will be fulfilled by the lead contact, Prof. George K. Tofaris (george.tofaris@ndcn.ox.ac.uk).

### Materials availability

This study did not generate new unique reagents.

### Data and code availability

• Data reported in this paper will be shared by the lead contact upon request.
• This paper does not report original code.
• Any additional information required to reanalyze the data reported in this paper is available from the lead contact upon request.

## Acknowledgments

We thank Dongnan Yan, MSc, Nuffield Department of Women’s and Reproductive Health, University of Oxford, for assistance. Graphical images were created with BioRender. The work was funded by a UKRI MRC Senior Clinical Fellowship (MR/V007068/1) to G.K.T. and grants from The Michael J. Fox Foundation, the Galen and Hilary Weston Foundation, the 10.13039/501100000272National Institute for Health and Care Research (NIHR) Oxford Biomedical Research Centre and UKRI MRC Developmental Pathway Funding Scheme (MR/Y019415/1) to G.K.T., the 10.13039/501100012166National Key Research and Development Program of China (2023YFC2307400), the 10.13039/501100001809National Natural Science Foundation of China (82472334 to B.S. and 82471500 to C.J.), the 10.13039/501100021171Guangdong Basic and Applied Basic Research Foundation (2022A1515010236 to B.S. and 2024A515012060 to C.J.), the Shenzhen International Science and Technology Cooperation Project (GJHZ20220913144002005), the 10.13039/501100004853Chinese University of Hong Kong startup funding (UDF02002573), the Pengcheng Peacock Research Fund (2024TC0150 and KQ002573), and the Shenzhen Peacock Team (KQTD20240729102029016) to C.J. The Oxford Discovery cohort is funded by a Cohort Studies Grant Award from 10.13039/501100000304Parkinson's UK and supported by the 10.13039/501100013373NIHR Oxford Biomedical Research Centre and the NIHR Clinical Research Network. The views expressed are those of the author(s) and not necessarily those of the 10.13039/100030827NHS, the 10.13039/501100000272NIHR, or the Department of Health or other funding bodies. For the purpose of open access, the authors have applied a CC BY public copyright license to any author accepted manuscript version arising from this submission.

## Author contributions

S.Y., W.Z., C.J., B.S., and G.K.T. designed the research. S.Y., W.Z., X.L., S.D., A.R.C., and A.S. performed the experiments and analyzed data. Y.L. assisted with experiments. C.L.L. and N.J.F.G. performed iPSC differentiation. M.T.H. provided clinical samples and information. C.L. interpreted data. C.J., B.S., and G.K.T. obtained funding, analyzed data, and provided experimental oversight. S.Y. and G.K.T. drafted the manuscript, with input from all of the authors.

## Declaration of interests

The authors declare no competing interests.

## STAR★Methods

### Key resources table

**Table:** 

REAGENT or RESOURCE	SOURCE	IDENTIFIER
**Antibodies**		

Anti-CD9 antibody (MM2/57)	Millipore	Cat# CBL162; RRID:AB_2075914
Anti-CD171 (L1CAM) antibody (UJ127)	Thermo Fisher Scientific	Cat# MA5-14140; RRID:AB_10979034
Anti-α-synuclein antibody (Syn1)	BD Biosciences	Cat# 610787; RRID:AB_398108
Biotin anti-CD81 antibody (M38)	Abcam	Cat# ab239238; RRID:AB_3665509
Biotin anti-CD11b antibody (ICRF44)	Thermo Fisher Scientific	Cat# 13-0118-82; RRID:AB_466368
Biotin anti-CD171 (L1CAM) antibody (UJ127)	Thermo Fisher Scientific	Cat# MA5-14137; RRID:AB_10990347
Biotin anti-CD56 (NCAM) antibody	Thermo Fisher Scientific	Cat# 13-0567-82; RRID:AB_10596499
Biotin anti-α-synuclein antibody (LB509)	BioLegend	Cat# 807710; RRID:AB_2832855
Biotin anti-α-synuclein aggregated antibody (A17183A)	BioLegend	Cat# 864906; RRID:AB_2832869
Anti-SDCBP (syntenin-1) antibody	Abnova	Cat# PAB7132; RRID:AB_1580423
Anti-CD81 antibody (EPR4244)	Abcam	Abcam Cat# ab109201; RRID:AB_10866464
Anti-CD63 antibody (Ts63)	Thermo Fisher Scientific	Cat# 10628D; RRID:AB_2532983
Anti-Flotillin-1 antibody	BD Biosciences	Cat# 610820; RRID:AB_398139
Anti-α-synuclein (phospho S129) antibody	Abcam	Cat# ab51253; RRID:AB_869973
Anti-Calnexin antibody	Abcam	Cat# ab22595; RRID:AB_2069006
Anti-GAPDH antibody	Abcam	Cat# ab9485; RRID:AB_307275
Anti-Tubulin β 3 (Tuj1) antibody	BioLegend	Cat# 801201; RRID:AB_2313773
Goat anti-mouse IgG (H + L) secondary antibody, HRP	Thermo Fisher Scientific	Cat# 31430; RRID:AB_228307
Goat anti-rabbit IgG (H + L) secondary antibody, HRP	Thermo Fisher Scientific	Cat# 31460; RRID:AB_228341
Alexa Fluor 647 anti-CD9 antibody	EXBIO Praha	Cat# A6-208-T100; RRID:AB_10733480
FITC anti-CD81 antibody	Thermo Fisher Scientific	Cat# A15753; RRID:AB_2534533
Anti-α-synuclein antibody (MJFR1)	Abcam	Cat# ab138501; RRID:AB_2537217
Anti-aggregated-α-synuclein antibody (5G4)	Millipore	Cat# MABN389; RRID:AB_2716647
Anti-CD171 (L1CAM) antibody (5G3)	Thermo Fisher Scientific	Cat# 14-1719-82; RRID:AB_891383
Anti-Tetraspanin Trio antibody	ONI (from EV profiler kit 2)	Cat# 800-00113
Anti-MAP2 antibody	Abcam	Cat# ab5392; RRID:AB_2138153
Goat anti-mouse IgG (H + L) secondary antibody, Alexa Fluor 568	Thermo Fisher Scientific	Cat# A-11004; RRID:AB_2534072
Goat anti-chicken IgY (H + L) secondary antibody, Alexa Fluor 647	Thermo Fisher Scientific	Cat# A-21449; RRID:AB_2535866

**Bacterial and virus strains**		

pLV-eGFP	Gift from Pantelis Tsoulfas	RRID:Addgene_36083
psPAX2	Gift from Didier Trono	RRID:Addgene_12260
pMD2.G	Gift from Didier Trono	RRID:Addgene_12259

**Biological samples**		

Human serum samples	This study	N/A

**Chemicals, peptides, and recombinant proteins**		

LDN193189 hydrochloride	Sigma	Cat# SML0559
A83-01	Tocris	Cat# 2939
Recombinant mouse sonic hedgehog/Shh (C25II)	R&D Systems	Cat# 464-SH-200
Purmorphamine	Tocris	Cat# 4551
Recombinant human FGF-8a protein	Tocris	Cat# 4745-F8
CHIR 99021	Tocris	Cat# 4423
Human/mouse/rat BDNF recombinant protein	PeproTech	Cat# 450-02
Human GDNF recombinant protein	PeproTech	Cat# 450-10
Human TGF-beta 3 recombinant protein	Thermo Fisher Scientific	Cat# PHG9305
DAPT	Tocris	Cat# 2634
L-Ascorbic acid	Sigma	Cat# A4403
Dibutyryl cAMP sodium salt	Sigma	Cat# AD0627
Mouse laminin	Sigma	Cat# L2020
MES hydrate	Sigma	Cat# M8250
N-(3-Dimethylaminopropyl)-N′-ethylcarbodiimide hydrochloride (EDC)	Sigma	Cat# 03450
N-Hydroxysuccinimide (NHS)	Sigma	Cat# 130672
TWEEN® 20	Sigma	Cat# P7949
Streptavidin β-galactosidase (SβG) conjugate	Thermo Fisher Scientific	Cat# S931
Mineral oil	Sigma	Cat# M5904
Triton™ X-100	Sigma	Cat# T8787
Fluorescein Di-β-D-Galactopyranoside (FDG)	Thermo Fisher Scientific	Cat# F1179
Glycine	Sigma	Cat# G7126
Tris buffer	Sigma	Cat# 648314
Paraformaldehyde	Sigma	Cat# 158127
2% uranyl acetate solution	Electron Microscopy Sciences	Cat# 22400-2
Polybrene®	Santa Cruz Biotechnology	Cat# sc-134220

**Critical commercial assays**		

Pierce™ BCA Protein Assay Kit	Thermo Fisher Scientific	Cat# 23225
U-PLEX human α-synuclein kit	Meso Scale Discovery	Cat# K151WKK-2
R-PLEX human CD81 (EV) assay	Meso Scale Discovery	Cat# K1515NR-2

**Experimental models: Cell lines**		

Human induced pluripotent stem cell (iPSC)	This study	N/A
SH-SY5Y	ATCC	RRID:CVCL_0019

**Software and algorithms**		

AutoCAD	Autodesk	https://www.autodesk.com/products/autocad/overview?term=1-YEAR; RRID:SCR_021072
NIS-Elements	Nikon	https://www.nikoninstruments.com/Products/Software; RRID:SCR_014329
Fiji ImageJ	GNU General Public License	https://ImageJ.net/software/fiji; RRID: SCR_002285
CODI	ONI	https://alto.codi.bio/
GraphPad Prism	GraphPad Software	http://www.graphpad.com/; RRID:SCR_002798
Biorender	Biorender	www.biorender.com; RRID:SCR_018361

**Other**		

mTeSR™1	Stem Cell Technologies	Cat# 85857
Versene solution	Thermo Fisher Scientific	Cat# 15040066
Geltrex™ LDEV-free reduced growth factor basement membrane matrix	Thermo Fisher Scientific	Cat# A1413202
DMEM - high glucose	Sigma	Cat# D5671
Fetal Bovine Serum (FBS)	Sigma	Cat# F7524
MEM Non-Essential Amino Acids Solution (100X)	Thermo Fisher Scientific	Cat# 11140050
GlutaMAX™ Supplement	Thermo Fisher Scientific	Cat# 35050061
Antibiotic-Antimycotic (100X)	Thermo Fisher Scientific	Cat# 15240062
TrypLE™ Express Enzyme (1X), phenol red	Thermo Fisher Scientific	Cat# 12605036
Halt™ protease and phosphatase inhibitor cocktail	Thermo Fisher Scientific	Cat# 78444
Encapsulated superparamagnetic microspheres polymer-COOH (Glacial Blue)	Bangs Laboratories	Cat# MEGB002
Encapsulated superparamagnetic microspheres polymer-COOH (Flash Red)	Bangs Laboratories	Cat# MEFR002
Bovine serum albumin (BSA)	Sigma	Cat# A7030
EZ-Link™ Micro Sulfo-NHS-LC-Biotinylation Kit	Thermo Fisher Scientific	Cat# 21935
SU-8 3025 Permanent negative epoxy photoresist	Kayaku	Cat# SU-8 3025
SYLGARD™ 184 Silicone Elastomer Kit	Dow-Corning	Cat# PDMS184
RIPA lysis and extraction buffer	Thermo Fisher Scientific	Cat# 89901
Amersham ECL prime western blotting detection reagent	Cytiva	Cat# RPN2232
HBSS	Thermo Fisher Scientific	Cat# 88284
Alexa Fluor® 647Conjugation Kit (Fast) - Lightning-Link®	Abcam	Cat# 269823
Alexa Fluor® 488 Conjugation Kit (Fast) - Lightning-Link®	Abcam	Cat# 236553
NEBuilder® HiFi DNA Assembly Master Mix	New England Biolabs	Cat# E2621S

### Experimental models and study participant details

#### Ethics statement

Sample collection for the Oxford Discovery cohort was approved by the South Central Oxford A Research Ethics Committee (IRAS 188167), written consent was obtained from all subjects, and all protocols followed the principles of the Declaration of Helsinki.

#### Clinical samples

Blood samples from the Oxford Discovery cohort were collected during the patient assessment, and the serum was isolated, aliquoted, and frozen at −80°C until further use. All samples were processed in a blinded fashion. A total of 60 subjects were included in this study. Serum samples and clinical data were collected from age- and gender-matched subjects, including individuals with iRBD (*n* = 20) or PD (*n* = 20), and HC (*n* = 20) as summarized in Table 1. The Oxford Discovery cohort is a prospective cohort of patients with iRBD and PD who were recruited from 11 hospitals in the Thames Valley region between September 2010 and September 2023. Inclusion criteria for iRBD were confirmation by video-assisted polysomnography and available serum samples. Participants were excluded if a secondary cause for RBD was present. Patients diagnosed with PD, and HC without significant comorbidities or relevant family history were included. Full details of the discovery cohort and full inclusion/exclusion criteria have been published elsewhere.^39^ There was no effect of age or sex on L1EV membrane-associated α-synuclein in tested subjects.

#### Generation of human iPSC-derived midbrain dopaminergic neurons

Dopaminergic neurons were derived from human iPSC lines using a previously published differentiation protocol optimized in-house.^26^^,^^40^ The ND34391G iPSC line (obtained from the Coriell Institute, distributed through the NINDS Fibroblasts and iPSCs Collection) was derived from a 55-year-old white female PD patient carrying a triplication of a large 1.5 Mb region including the *SNCA* gene and the corresponding *SNCA* isogenic clone was previously generated from this line (Table S2).^41^ Briefly, human iPSC lines were maintained in mTeSR1 media (85857, Stem Cell Technologies) and routinely screened for mycoplasma contamination prior to differentiation. The cells were passaged when reaching 90–95% confluency using Versene (15040066, Gibco) onto Geltrex (A1413202, Thermo Fisher Technologies) coated plates until they achieved the appropriate morphology and confluency for differentiation. Human iPSCs were directed toward a midbrain floor plate lineage over 12 days using 100 nM LDN193189 (SML0559, Sigma), 2 μM A83-01 (2939, Tocris), 300 ng/mL Sonic Hedgehog (464-SH-200, R&D Systems), 2 μM purmorphamine (4551, Tocris), 200 ng/mL FGF8α (4745-F8, R&D Systems), and 3 μM CHIR99021 (4423, Tocris). Following this stage, the cells were further differentiated into a dopaminergic neuron lineage from day 21 onward using 20 ng/mL BDNF (450-02, PeproTech), 20 ng/mL GDNF (450-10, PeproTech), 1 ng/mL TGFβ3 (PHG9305, Thermo Fisher Scientific), 10 μM DAPT (2634, Tocris), 200 μM ascorbic acid (A4403, Sigma), 500 μM db-cAMP (D0627, Sigma), and 1 μg/mL laminin (L2020, Sigma).

#### Cell line cultures

SH-SY5Y neuroblastoma cells (human, female) were originally obtained from the European Collection of Authenticated Cell Cultures (RRID:CVCL_0019) and were maintained in high-glucose DMEM (D5671, Sigma) supplemented with 10% (v/v) FBS (F7524, Sigma), 1X MEM non-essential amino acids (11140035, Thermo Fisher Scientific), 1X GlutaMAX (35050061, Thermo Fisher Scientific), and 1X Antibiotic-Antimycotic (15240062, Thermo Fisher Scientific). Cells were grown at 37°C in 5% CO2 at 95% humidity and were detached using TrypLE (12605036, Thermo Fisher Scientific) for routine subculturing.

### Method details

#### EV isolation from serum

To isolate EVs from serum, a three-step sequential centrifugation (300 g for 10 min, 2,000 g for 20 min, and 10,000 g for 30 min) was first applied to remove cellular debris, protein aggregates and fatty material from the sample. The supernatant, i.e., pre-cleared serum, was transferred to protein low-binding tubes (0030108450, Eppendorf) for further processing as follows:

For SEC, we used the HiScreen Capto Core 700 prepacked filtration column (17548115, Cytiva) to separate EVs from the bulk of free serum proteins as previously described.^20^ In brief, 1 mL of pre-cleared serum was loaded onto the SEC column and 10 fractions were collected in PBS at a flow rate of 1 mL/min.

For immunocapture, 10-fold excess of DAPI- (MEGB002, Bangs Laboratories) or Cy5- (MEFR002, Bangs Laboratories) labeled carboxylated magnetic encapsulated beads relative to the number of EVs, as determined by NTA, were transferred into a protein low-binding tube and washed three times with 500 μL of 2-morpholinoethanesulfonic acid (MES, A8250, Sigma) buffer (50 mM, pH 5.5). Freshly prepared 10 mg of 1-ethyl-3-(3-(dimethylamino)propyl) carbodiimide hydrochloride (EDC, 03450, Sigma) and 10 mg of N-hydroxysuccinimide (NHS, 130672, Sigma) were reconstituted in 1 mL of MES buffer just prior to use. To activate the beads, 500 μL of the mixture was added to the beads and incubated for 1 h at room temperature with rotation. The activated beads were then washed twice with 500 μL of PBS, followed by adding 5 μg of capture antibody per mg beads. The capture antibodies against the following markers were conjugated to the beads: CD9 (CBL162, Millipore), or L1CAM (UJ127: MA5-14140, Thermo Fisher Scientific), or α-synuclein (Syn1: 610787, BD Biosciences). Activated beads and antibodies were incubated for 1.5 h with rotation. The antibody-conjugated beads were then washed twice with 500 μL of PBS followed by blocking with 500 μL of 1% Bovine Serum Albumin (BSA, A7030, Sigma) in PBS for 1 h with rotation. Beads without antibody conjugation (beads alone) were included as a negative control. The beads were washed twice with 500 μL of PBS and resuspended in 1 mL of PBS containing protease and phosphatase inhibitor cocktail (78444, Thermo Fisher Scientific). The antibody-coated beads were incubated with either 20 μL of pre-cleared serum or 40 μL of EV-enriched SEC F3+F4 at 4°C overnight with rotation. The supernatant of 20 μL of serum, collected after ultracentrifugation (100,000 g for 90 min), i.e., EV depleted serum was included as a negative control. The bead-EV complexes were collected by magnetic separation and washed successively with PBS containing 0.05% Tween 20 (P7949, Sigma) and PBS for downstream analysis.

#### EV isolation from CM

One mL of SH-SY5Y cell culture media, conditioned for 48 h, was collected from a well of a 12-well plate (400,000 cells per well), aliquoted in protein low-binding tubes, and serially centrifuged at 300 g for 10 min, followed by 2,000 g for 10 min and 10,000 g for 30 min all at 4 °C. The resulting supernatant (i.e., pre-cleared CM) was stored at −80°C. Thirty μL of pre-cleared CM was processed for immunocapture of L1EVs as described above for serum or subjected to NTA, and 1 mL of pre-cleared CM was passed through PES filters (0.22 μm, SLGP033RS, Millipore) before total EV isolation using ultracentrifugation (100,000 g for 90 min at 4 °C).

For iPSC-derived cultures, 500 μL of neuronal media was conditioned from Day 52 to Day 60 and collected from a well of a 12-well plate (1.0×10^6^ neurons per well). Media from one differentiation were pooled and considered one biological replicate. We chose this time-period because we previously showed that iPSC-derived dopaminergic neurons are electrophysiologically active from D45 onwards.^40^ The CM were pre-cleared and stored as described above for SH-SY5Y cells. Thirty μL of pre-cleared CM was processed for immunocapture with anti-L1CAM coated beads as described above or subjected to NTA. For SEC, 4 mL pre-cleared CM was passed through HiScreen Capto Core 700 prepacked filtration, and EV-enriched fractions were collected as described above for serum.

#### Labeling of EV-bead immunocomplexes for surface marker analysis

To visualise relevant surface markers on the EV-bead immunocomplexes, 200 μL of 0.1 μg/mL biotinylated detection antibody was added to the isolated EVs and incubated for 1 h at room temperature with rotation. Biotinylated antibodies against the following markers were used: CD81 (ab239238, Abcam), CD11b (13-0118-82, Thermo Fisher Scientific), L1CAM (MA5-14137, Thermo Fisher Scientific), NCAM (13-0567-82, Thermo Fisher Scientific), α-synuclein (LB509: 807710, BioLegend), aggregated α-synuclein (A17183A: 864906, BioLegend), syntenin-1 (PAB7132, Abnova). For anti-syntenin-1, antibody conjugation to biotin was performed using a commercial conjugation kit (21935, Thermo Fisher Scientific). The resultant EV-bead immunocomplexes were separated by a magnet and the supernatant was removed. Following three washes with PBS, 200 μL of 2 μg/mL SβG (S931, Thermo Fisher Scientific) was mixed with the bead-EV immunocomplexes and incubated for 30 min at room temperature with rotation. The enzyme-labelled immunocomplexes were washed three times with PBS and resuspended to a final concentration of 1.4×10^7^ beads/ml with PBS for loading onto the microfluidic platform.

#### Microfluidic chip design and fabrication

We designed a pump-free, syringe-vacuum-driven, droplet-based microfluidic chip with dual inlets for independent introduction of the pre-conjugated enzyme-labelled immunocomplexes and fluorescein substrate (FDG). This design enables simple and high-throughput microdroplet generation followed by the self-assembling of a high-density monolayer droplet array for *in situ* incubation and fluorescence imaging (Video S1). The microfluidic chip comprises a pair of oil inlets, one inlet for the introduction of the EV-bead immunocomplex and the other for the introduction of the fluorescein substrate solution, a flow-focusing structure for droplet generation, a Christmas tree-shaped network of distribution channels, and a collection chamber enabling self-assembly of the monolayer droplet array. Once the sample and the substrate are loaded into the inlet ports, the fluids are driven by withdrawing the piston of the connecting syringe to a pre-determined pressure differential across the chip. The microfluidic chip is made of a single polydimethylsiloxane (PDMS) layer irreversibly bonded to a glass slide. The PDMS structure was fabricated using a refined soft lithography technique. The microchannel patterns (Figure S1) were designed using AutoCAD software (Autodesk), which were then transferred to a high-resolution photomask. SU-8 (SU-8 3025, Kayaku) master molds were created with a channel depth of 25 μm, adhering to manufacturer’s guidelines. Post-master mold preparation, a 1:8 mixture of PDMS curing agent and elastomer (PDMS184, Dow-Corning) was degassed, poured over the SU-8/Si master (LY-118-A-1315, Youli Biotech), and cured in an oven at 120°C for 20 min. The cured PDMS was then peeled off and its inlet and outlet holes were punched. Afterward, the PDMS layer was bonded to a glass slide (75 × 25 × 1.1 mm) or a square glass substrate (70 mm × 70 mm × 1 mm) in the case of eight independent assay units, using oxygen plasma treatment for 20 s. The assembled droplet-based microfluidic chip was further cured in an oven overnight at 80°C to strengthen the bond.

#### Droplet generation

Prior to droplet generation, the microfluidic chip was filled with an oil phase (mineral oil, [M5904, Sigma] containing the stabilising surfactants 3% ABIL EM 90 [Degussa] and 0.1% w/w Triton X-100 [T8787, Sigma]). Then, a 5 mL single-use plastic syringe was connected to the outlet of the chip with a length of Teflon tubing (0.46 mm × 0.76 mm, [i.d. × o.d.]). When the capillary force-driven oil flows reach the sample inlet, 10 μL of enzyme-labelled EV-bead immunocomplexes and 10 μL of FDG (F1179, Thermo Fisher Scientific) substrate solution were individually loaded into the two sample inlets with a micropipette, ensuring no air-bubbles were trapped in the inlets. To initiate the droplet generation, the piston of the syringe was pulled outward and locked in place at an approximately 1 mL volume using a binder clip. Once the collection chamber was filled with the microdroplets, the piston was gently pushed back to its starting position, and then the tubing was cut off.

#### Fluorescence image acquisition

After incubation for 10 min at room temperature, the microfluidic chip was subjected to continuous imaging via an inverted fluorescence microscope (Eclipse Ti2, Nikon). This setup, equipped with a 10× magnification lens, allowed for the capture of an area of 953× 1432 μm^2^ per frame, accommodating approximately 2,500–3,500 microdroplets. Brightfield images cataloged the total droplet count, while fluorescence images (DS-Ri2, Nikon) identified the total fluorescently-labelled bead count and the fluorescein (green fluorescence)-positive droplets with a signal-to-noise ratio exceeding 3. For detailed size analysis, three distinct regions were imaged at 10× magnification, and measurements of over 10,000 droplets were conducted using NIS-Elements (Nikon) software.

#### Image analysis

The number of droplets was counted using brightfield images and a watershed algorithm was applied to separate any partially connected droplets. Each bead was identified, and by setting the intensity threshold, the number of fluorescently labeled beads (Cy5 or DAPI) was counted using the Find Maxima function. Then, the number of positive droplets was counted using the fluorescent images captured in the FITC fluorescence channel. A background correction algorithm was then applied to the image to subtract the mean intensities of the droplets that did not contain a bead. If a fluorescent droplet was not colocalised with a bead, the droplet was excluded from analysis. Using this approach, the total number of fluorescent droplets over the total number of beads was calculated. Twenty fields of view were acquired for quantitative analysis. Image analysis was performed using ImageJ 1.53t (Fiji) software.

#### Immunoblotting

EVs isolated using SEC from 1 mL of pre-cleared serum or by immunocapture from 500 μL of pre-cleared serum, 1mL of SEC F3+F4 CM, or 4 mL of pre-cleared CM, were lysed in 20 μL of ice-cold radioimmunoprecipitation assay (RIPA) buffer (89901, Thermo Fisher Scientific) supplemented with protease and phosphatase inhibitor cocktail. The lysates were collected after incubation for 30 min at room temperature with shaking. The protein concentration was measured with a BCA assay (23225, Thermo Fisher Scientific). The denatured and reduced samples were resolved by SDS–polyacrylamide gel electrophoresis (PAGE) using NuPAGE 4 to 12% bis-tris gels and transferred to nitrocellulose membrane using the iBlot 2 Gel Transfer system (IB21001, Thermo Fisher Scientific). The membranes were incubated with primary antibodies overnight at 4°C. The following primary antibodies were used for immunoblots: anti-CD81 (1:1000, ab109201, Abcam), anti-CD63 (1:1000, 10628D, Thermo Fisher Scientific), anti-CD9 (1:1000, CBL162, Millipore), anti-Flotillin-1 (1:1000, 610820, BD Biosciences), anti-L1CAM (1:1000, MA5-14140, Thermo Fisher Scientific), anti-α-synuclein (1:1000, 610787, BD Biosciences), anti-phosphoSer129 α-synuclein (1:2000, ab51253, Abcam), anti-Calnexin (1:2000, ab22595, Abcam), anti-GAPDH (1:2000, ab9485, Abcam), and anti-Tuj1 (1:10000, 801201, BioLegend). The relevant protein bands were detected using horseradish peroxidase-conjugated goat anti-mouse IgG (1:5000, 31430, Thermo Fisher Scientific) or horseradish peroxidase-conjugated goat anti-rabbit IgG (1:5000, 31460, Thermo Fisher Scientific), followed by Amersham enhanced chemiluminescence prime detection reagent (RPN2232, Cytiva). Blots were imaged on a Bio-Rad ChemiDoc imaging system.

#### NTA

To measure EV concentrations, either pre-cleared serum, pre-cleared CM, or EVs eluted from beads, were serially diluted in PBS (1:100 to 1:20,000) to bring the concentration of EVs within the operating dynamic range. The appropriate dilution was injected into the NTA observation chamber using a syringe. Videos capturing the Brownian motion of particles were recorded to determine the number and size distribution of EVs. NTA was conducted using an NS300 nanoparticle analyser (NanoSight). Camera settings were maintained between levels 8–12 with automatic adjustments for all post-acquisition parameters except for the detection threshold, which was set at 5. For each sample, three 30-s videos were recorded with a 5-s intermission, using the script control function.

#### TEM

TEM was used to examine the shape, size and morphology of EVs. Specifically, EVs isolated from 1 mL of serum or 2 mL of CM using either anti-CD9 or anti-L1CAM-coated beads, were eluted by adding 25 μL of glycine (G7126, Sigma) solution (100 mM, pH 2.8) to the beads, followed by gentle shaking for 30 min at room temperature. The pH was adjusted back to neutral using Tris buffer (648314, Sigma), followed by adding 2 μL of 4% paraformaldehyde (PFA, 158127, Sigma). Ten μL of resultant eluent samples was applied to freshly glow discharged carbon formvar 400 mesh copper grids (D400FC, Electron Microscopy Sciences) for 50 min. The grid was blotted with filter paper from the side and stained with 2% uranyl acetate (UA, 22400-2, Electron Microscopy Sciences) for 10 s. Next, extra UA was blotted dry and air dried. The EVs on grid were imaged with a TEM (JEM-1400Flash, JEOL) operated at 120 kV using a Gatan Rio CMOS camera.

#### NanoFCM

EV surface markers CD81 and CD9 were analyzed by NanoFCM using a NanoAnalyzer U30 instrument (NanoFCM Inc.) with dual 638/488 nm lasers. Total EVs isolated from 1 mL of serum, using ultracentrifugation, were resuspended in 1% BSA in PBS containing protease inhibitor cocktails. The EVs were stained with an antibody cocktail solution consisting of Alexa Fluor 647 labeled anti-CD9 (A6-208-T100, EXBIO Praha) and FITC labeled anti-CD81 (A15753, Thermo Fisher Scientific) at 4°C overnight in the dark. Post-incubation, EV samples were diluted in PBS for immediate phenotypic analysis. Data were processed using NanoFCM Professional Suite v2.0 software (NanoFCM Inc.).

#### dSTORM

EVs isolated from serum using SEC or from CM using ultracentrifugation were resuspended in 0.5% BSA in HBSS buffer (88284, Thermo Fisher Scientific) containing protease and phosphatase inhibitor cocktails. Anti-α-synuclein (MJFR1: ab138501, Abcam) and anti-aggregated-α-synuclein (5G4: MABN389, Millipore) antibodies were labeled with Alexa 647 using the Alexa Fluor 647 conjugation kit (ab269823, Abcam), and anti-L1CAM antibody (5G3: 14-1719-82, Thermo Fisher Scientific) was labeled with Alexa 488 using the Alexa Fluor 488 conjugation kit (ab236553, Abcam) following manufacturer’s protocol. Serum EVs were stained with a cocktail of anti-α-synuclein-Alexa Fluor 647, anti-L1CAM-Alexa 488, and Tetraspanin Trio-Alexa 561 (800-00113, ONI) at 4°C overnight in the dark. EVs from CM were stained with an antibody cocktail consisting of anti-aggregated-α-synuclein-Alexa Fluor 647 and Tetraspanin Trio-Alexa 561 at 4°C overnight in the dark. Following staining, EV samples were immobilized on 170 μm (#1.5H) thick 3% BSA pre-coated glass slides for 1.5 h at 4°C in the dark, washed 10 to 12 times with PBS to remove any floating particles and free antibodies, followed by the addition of dSTORM imaging buffer (900-00083, ONI). Super-resolution microscopy images of labeled EVs were acquired using ONI Nanoimager (Oxford Nanoimaging) equipped with a 100×, 1.4 numerical aperture oil immersion objective and NimOS software (version 1.7). A total of 7,500 frames (2,500 per channel) were recorded for localization mapping using 30% power on each of the 488-, 561-, and 647-nm lasers, respectively. Colocalization analysis of different molecules on single EVs was analyzed using CODI online platform (https://alto.codi.bio/). Nine fields of view were acquired for quantitative analysis.

To identify single EVs on beads, EVs immunocaptured on anti-L1CAM coated DAPI-labelled beads from serially diluted serum at 1:10, 1:1, or 1:0.1 (bead:EV) ratio were stained with anti-CD9-Alexa Fluor 647 or Tetraspanin Trio-Alexa 561 at 4°C overnight in the dark. The EV depleted serum was included as a negative control. Following three washes with 1 mL of PBS, beads were mounted on 170 μm (#1.5H) thick glass slides and images were acquired as described above.

#### MSD electrochemiluminescence

L1EVs immunocaptured from 250 μL of pre-cleared serum, or total EVs enriched in SEC F3+F4 from 1 mL of serum or from 4 mL of CM, were lysed in PBS containing 1% Triton X-100, supplemented with protease and phosphatase inhibitor cocktail. The lysates were collected after incubation for 30 min at room temperature with shaking. α-Synuclein and CD81 levels in the lysates or 4-fold diluted serum were measured using MSD electrochemiluminescence U-PLEX assay (K1515NR-2, Meso Scale Discovery) and R-PLEX assay (K1515NR-2, Meso Scale Discovery), respectively.

#### Molecular cloning and lentivirus production

NEBuilder HiFi DNA Assembly Master Mix (NEB, E2621S) was used to combine two PCR fragments with homologous ends, one generated from pLV-eGFP (gift from Pantelis Tsoulfas; 36083, Addgene) and consisting of the blasticidin S deaminase coding sequence preceded by the UbC promoter, and the other generated from a pre-existing lentiviral transfer plasmid and consisting of the CMV promoter/enhancer, the *SNCA* coding sequence and sequences required for lentivirus production. The combined vector, pLenti-CMV-WTαSyn-BlastR, was used to generate additional A53T, E46K, G51D, A30P, 3K (E35K + E46K + E61K), and 3D (V40D + G51D + V66D) α-synuclein-encoding lentiviral transfer plasmids via HiFi Assembly with mutagenic primers. The correct DNA sequences were confirmed by Sanger sequencing. Lentiviruses were prepared by transfecting HEK293T cells with the packaging plasmid psPAX2 (gift from Didier Trono; 12260, Addgene), the envelope plasmid pMD2.G (gift from Didier Trono; 12259, Addgene), and a transfer plasmid generated as described above (1:1:1 M ratio), followed by medium replacement on the following day and CM collection after a further two days. CM were spun at 300 x g for 5 min to pellet debris, and the supernatants were passed through PES filters (0.22 μm) before use.

#### Lentiviral transduction of SH-SY5Y cells and cell fractionation

SH-SY5Y cells were reverse transduced with lentiviruses in 12-well plates in a culture medium consisting of antibiotic-free high-glucose DMEM supplemented with 5% (v/v) FBS, 1X MEM non-essential amino acids, 1X GlutaMAX and 5 μg/mL polybrene (sc-134220, Santa Cruz Biotechnology). The following day, the lentivirus-containing medium was aspirated, the cells were rinsed once with PBS and then incubated in fresh high-glucose DMEM supplemented with 5% FBS, MEM non-essential amino acids, GlutaMAX and 1X Antibiotic-Antimycotic. After a further two days, the entire 1 mL of CM was collected.

Immediately after collecting the CM, cytosol-membrane fractionation was performed as described previously.^25^ Briefly, the cell monolayer was rinsed with PBS before addition of 100 μL of cytosol extraction buffer (10 mM PIPES, pH 7.4, 300 mM sucrose, 100 mM NaCl, 5 mM MgCl_2_, 5 mM EDTA, and protease and phosphatase inhibitors cocktail, supplemented with 900 μg/mL digitonin). After incubating for 15 min at 4°C with gentle agitation, the cytosolic fraction was collected into a 1.5 mL tube without disturbing the cell monolayer. Next, 100 μL of membrane extraction buffer was added (same as cytosol buffer except for supplementation with 0.5% Triton X-100 in place of the digitonin). After incubating for another 15 min at 4°C with gentle agitation, the membrane fraction was collected into a 1.5 mL tube, again without disturbing the cell monolayer. Cytosol and membrane fractions were both centrifuged at 1000 x g for 10 min at 4°C to pellet cell debris, and the supernatants were transferred to fresh tubes.

Separation of cell lysates into RIPA-soluble and insoluble fractions was performed as follows. First, the cell monolayer was rinsed with ice-cold PBS and lysed in 100 μL of ice-cold RIPA lysis buffer containing protease and phosphatase inhibitors cocktails. After incubating for 15 min at 4°C with gentle rocking, cell lysates were scraped into tubes, processed with a handheld homogenizer, and centrifuged at 1000 x g for 10 min at 4°C to pellet cell debris. The supernatants were transferred to ultracentrifuge tubes and spun at 100,000 x g for 1 h at 4°C. The soluble fractions were extracted and stored, while the RIPA-insoluble pellets were resuspended in the above lysis buffer with the SDS concentration adjusted to 2% (v/v).

#### Immunofluorescence staining

iPSC-derived neurons were fixed in 4% PFA/2% sucrose in PBS solution, permeabilised in 0.1% Triton X-100 in PBS, followed by blocking with 5% BSA in PBS. Cells were then incubated with primary antibodies, followed by fluorescently labeled secondary antibodies. The following primary antibodies were used: anti-α-synuclein (1:1000, 610787, BD Biosciences) and anti-MAP2 (1:2000, ab5392, Abcam). The following secondary antibodies were used: Alexa Fluor 568-labelled goat anti-mouse IgG (1:2000, A11004, Thermo Fisher Scientific) and Alexa Fluor 647-labelled goat anti chicken IgY (1; 2000, A21449, Thermo Fisher Scientific). Images were obtained using Zeiss LSM 980 Airyscan confocal microscope (Carl Zeiss AG). To quantify fluorescence intensity, for each differentiation, 3 images were obtained per condition and 25 to 40 cells per image were quantified.

### Quantification and statistical analysis

For multiple comparisons, we performed non-parametric statistical testing where the data were not normally distributed: Kruskal-Wallis one-way analysis of variance with the Dunn test for post hoc comparison between individual pairings when there were three or more independent groups. For normally distributed data, comparisons between two groups were analyzed using student’s two-tailed t test; comparisons among three or more groups were performed with ordinary one-way ANOVA using Prism 10.2.0 (GraphPad Software). Data from different groups were analyzed using receiver operating characteristic (ROC) with 95% CIs (2.5%–97.5%). Correlations between biomarkers were calculated using Spearman correlation as the data were not normally distributed. Values with *p* < 0.05 were regarded as significant.

## Supplemental information

Document S1. Figures S1–S10 and Tables S1 and S2

Document S2. Article plus supplemental information
