# Supplementary material for: Single extracellular vesicle detection assay identifies membrane-associated α-synuclein as an early-stage biomarker in Parkinson’s disease
Source: Cell Rep Med. 2025 Mar 7;6(3):101999. doi: 10.1016/j.xcrm.2025.101999 (PMC11970385; doi:10.1016/j.xcrm.2025.101999)
Supplement: Document S1. Figures S1–S10 and Tables S1 and S2 [file mmc1.pdf]

Cell Reports Medicine, Volume *6*

Supplemental information

Single extracellular vesicle detection assay identifies membrane-associated α-synuclein as an early-stage biomarker in Parkinson's disease

Shijun Yan, Wenjing Zhang, Xinying Li, Suman Dutta, Andrew R. Castle, Yiming Liu, Anis Sahoo, Chor Lai Lam, Nicholas J.F. Gatford, Michele T. Hu, Chen-zhong Li, Cheng Jiang, Bowen Shu, and George K. Tofaris

Supplemental information

Single extracellular vesicle detection assay identifies membrane-associated α-Synuclein as an early-stage biomarker in Parkinson's disease

Shijun Yan, Wenjing Zhang, Xinying Li, Suman Dutta, Andrew R. Castle, Yiming Liu, Anis Sahoo, Chor Lai Lam, Nicholas J. F. Gatford, Michele T. Hu, Chen-zhong Li，Cheng Jiang, Bowen Shu, and George K. Tofaris

Contents

Figure S1. Detailed mask design of the microfluidic chip. Related to Figure 1........................2

Figure S2. Robust droplet generation by using syringe-vacuum-driven microfluidic device. Related to Figure 1..3

Figure S3. Quantification of bead-to-droplet ratio according to Poisson distribution. Related to Figure 1..4

Figure S4. Time course of fluorescent signal generation through the microdroplet-confined enzymatic reaction. Related to Figure 1. 5

Figure S5. Confirmation of EV isolation from serum. Related to Figure 1..............................6

Figure S6. Nano flow cytometry analysis of membrane-associated CD9 and CD81 expression on serum single EVs. Related to Figure 2..7

Figure S7. Detection of α-synuclein and CD9 on serum L1EVs. Related to Figure 3. 8

Figure S8. Confirmation of EV isolation from conditioned media. Related to Figure 4...........9

Figure S9. L1EV membrane-associated α-synuclein increases under pathological conditions. Related to Figure 4...10

Figure S10. Reproducibility of the assay in detecting L1EV membrane-associated α-synuclein. Related to Figure 5. 11

Table S1. Performance of the assay in detecting serum EV membrane-associated α-Syn and CD81 following anti-L1CAM immunocapture. Related to Figure 3......................................12

Table S2. iPSC clones used in this study. Related to STAR Methods. 13

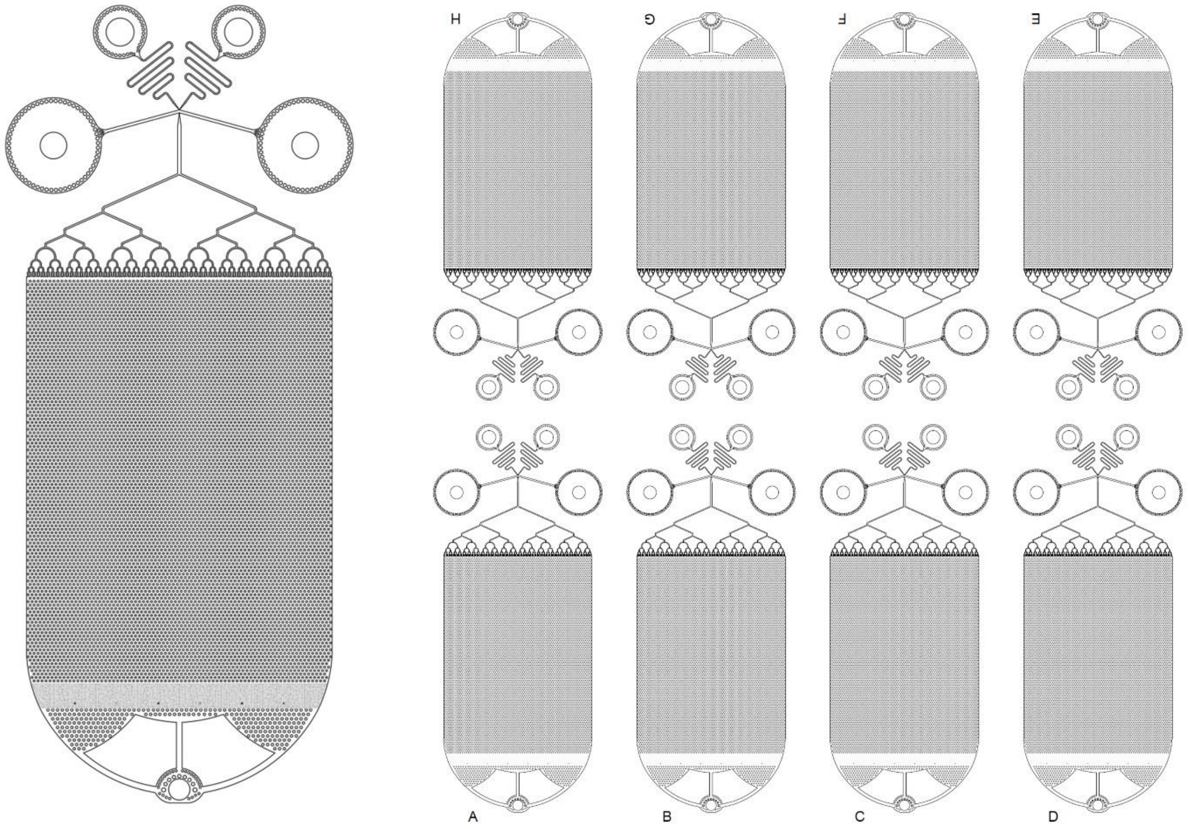

Figure S1. Detailed mask design of the microfluidic chip. Related to Figure 1.

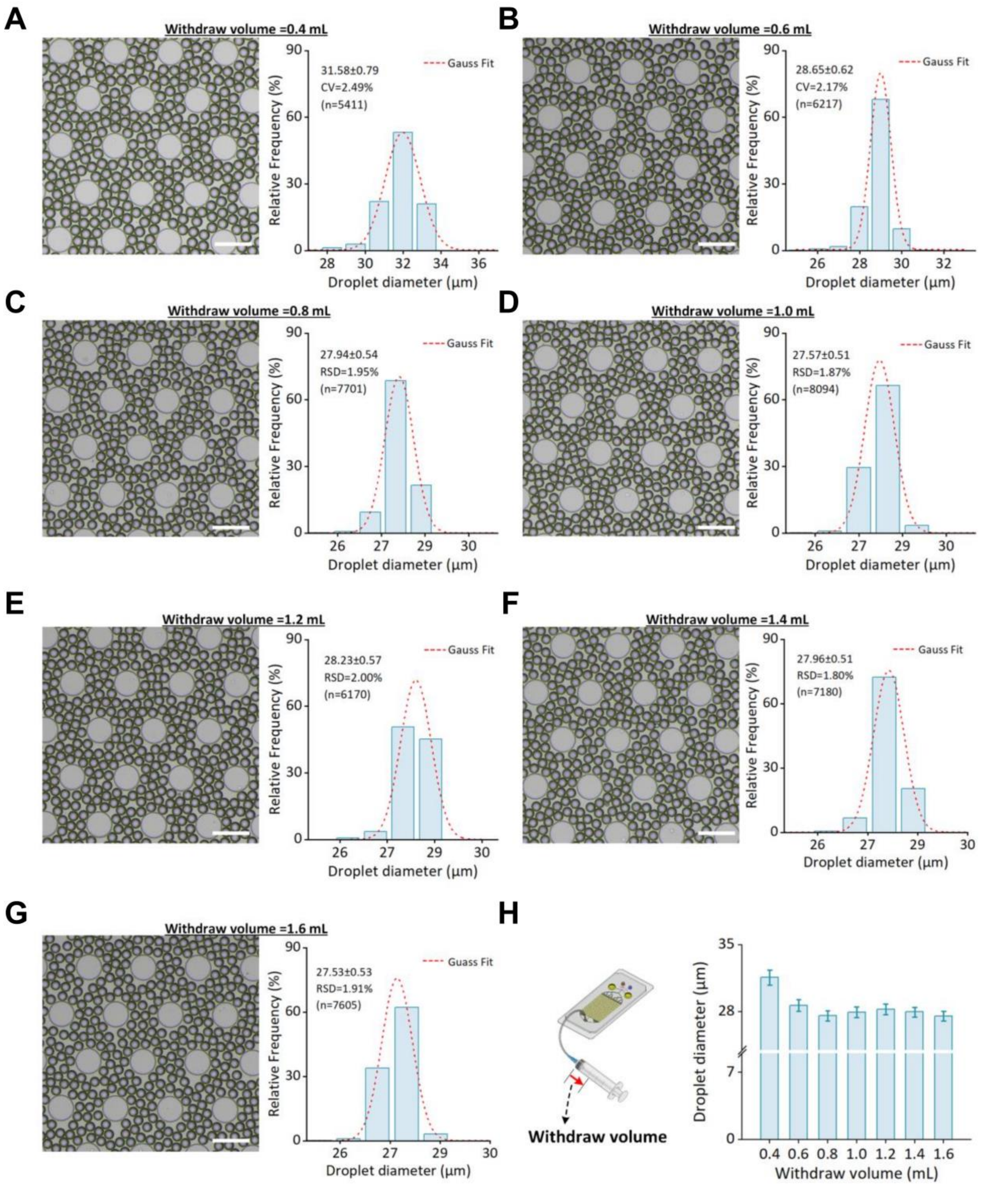

Figure S2. Robust droplet generation by using syringe-vacuum-driven microfluidic device. Related to Figure 1. (A to G) Microscopic images of droplets and the corresponding size distributions under different negative pressures, initiated by pulling the piston of a 5 mL plastic syringe outward and locking it at the place, with a piston withdrawal volume ranging from 0.4 mL to 1.6 mL (scale bar: 100 μm). **(H)** Schematic and summary of the syringe-vacuum-driven microfluidic device for highly uniform picolitre-sized droplet generation, demonstrating its capability as an easy-to-operate and robust microdroplet-based digital assay.

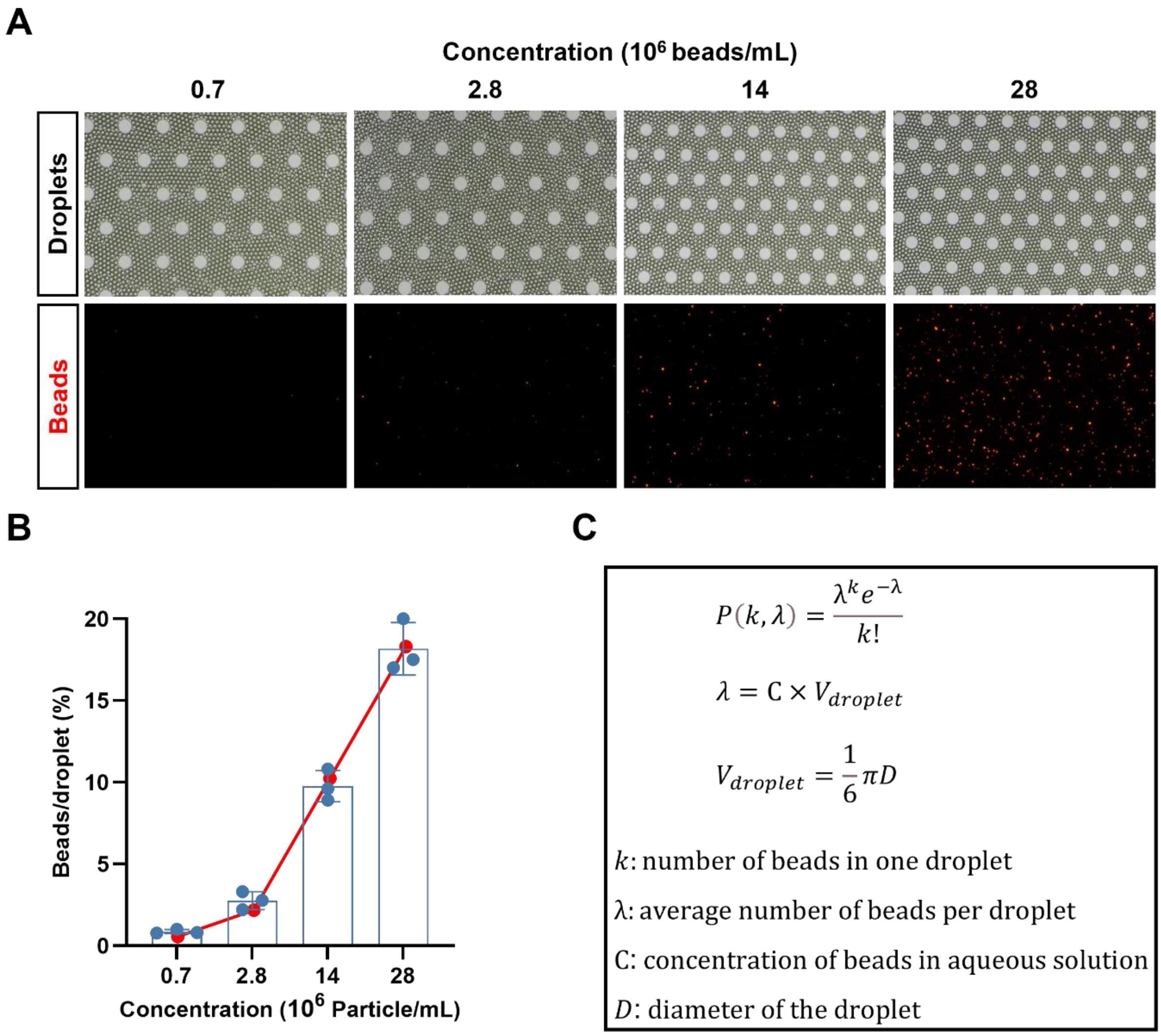

Figure S3. Quantification of bead-to-droplet ratio according to Poisson distribution. Related to Figure 1. (A) Representative images and **(B)** quantification of serial dilutions of input beads. The Poisson distribution (red line) predicts that at a concentration of 1.4×10^7 beads/mL, ~10.2% of droplets contain only one bead, which aligns with our analysis showing that 9.8% of droplets contain a single bead (blue histogram). **(C)** Theoretical calculations of Poisson distribution. n = 3 independent experiments. Data are represented as mean ± SD.

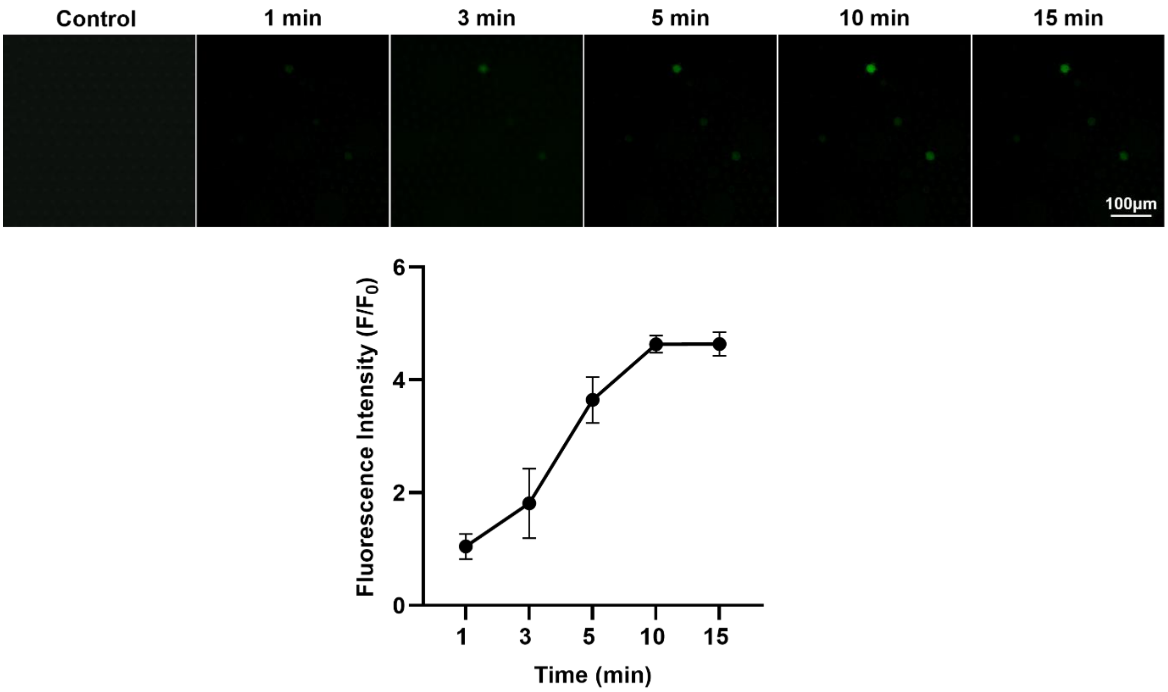

Figure S4. Time course of fluorescent signal generation through the microdroplet-confined enzymatic reaction. Related to Figure 1. Representative images of fluorescent droplets and relative fluorescence intensity in the fluorescent droplets over time during the enzymatic reaction process. Scale bar, 100 μm. Data are represented as mean ± SD.

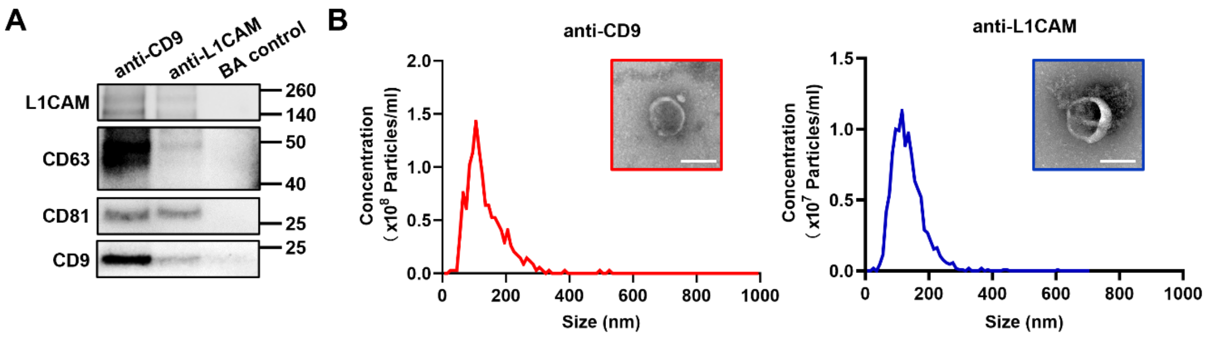

Figure S5. Confirmation of EV isolation from serum. Related to Figure 1. (**A**) Immunocapture using anti-CD9 or anti-L1CAM from neat serum followed by immunoblotting, with beads alone (BA) used as a control. (**B**) Size distribution of eluted EVs after anti-CD9 (red) or anti-L1CAM (blue) immunocapture was confirmed by NTA and the typical cap-shaped appearance was demonstrated by TEM. Scale bar, 100 nm.

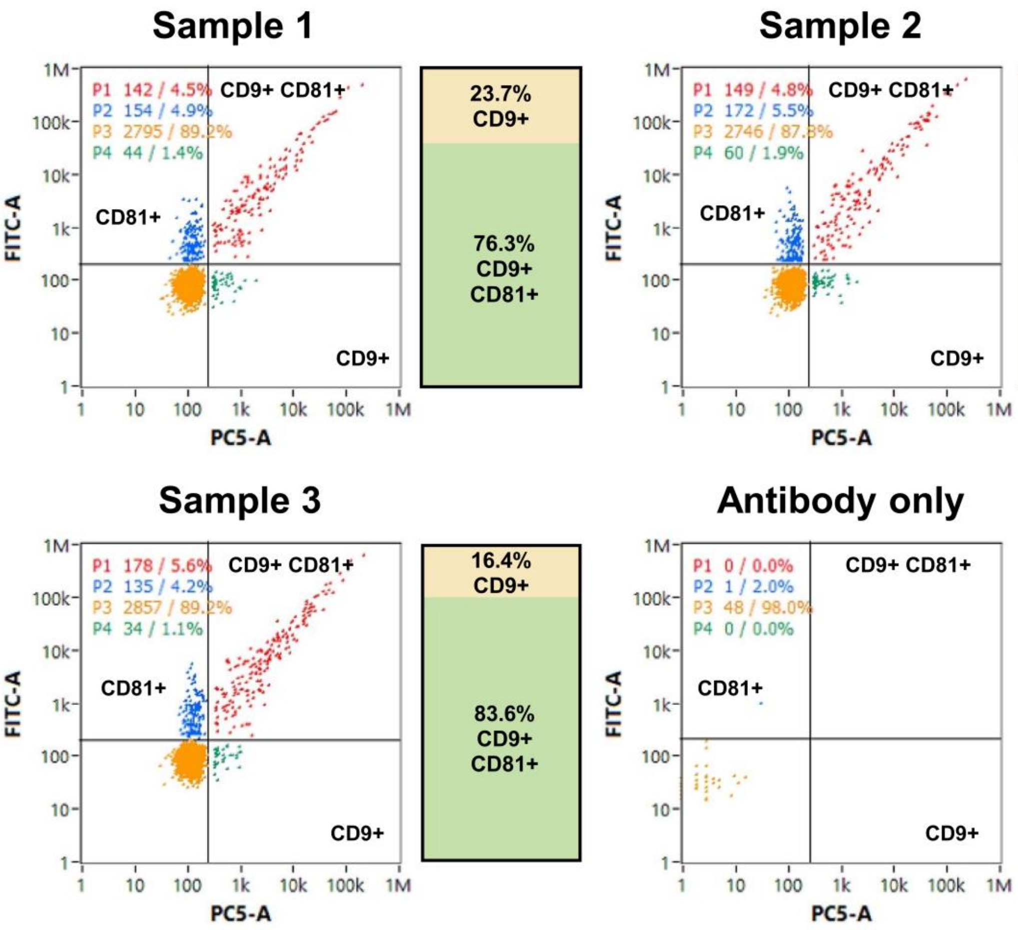

Figure S6. Nano flow cytometry analysis of membrane-associated CD9 and CD81 expression on serum single EVs. Related to Figure 2. Following ultracentrifugation, EVs (n = 3 individuals with Parkinson's disease tested) were stained with Alexa Fluor-647 labelled anti-CD9 and FITC labelled anti-CD81, and we also included antibody only (without sample) as a control. As previously reported (Xu et al., J Extracell Vesicles, 2024,3(10):p.e70016), the majority of EVs were unlabelled (orange) or not recognised as such by this method. Focusing on the labelled particles, we estimated that on average from the three samples tested 77.2% of CD9+ EVs were also CD81+, in agreement with estimates from our droplet-based assay (as summarised in **Figure 2A** and **2B**).

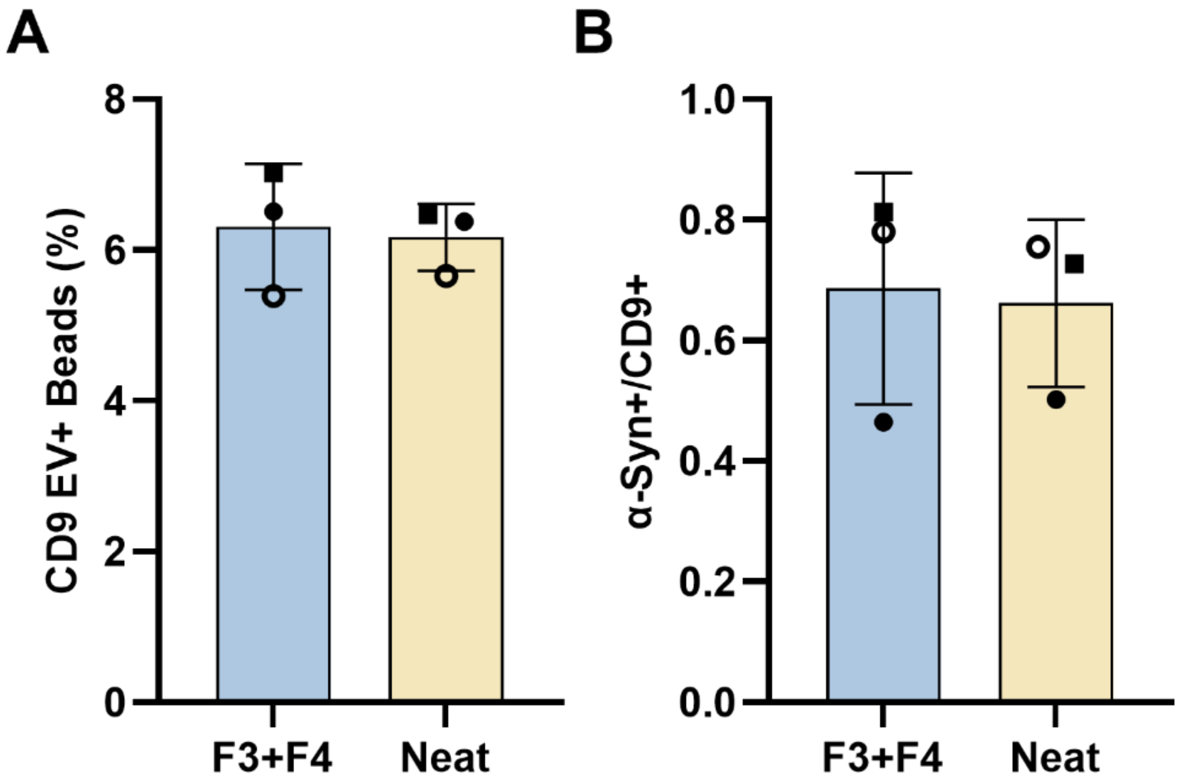

Figure S7. Detection of α-synuclein and CD9 on serum L1EVs. Related to Figure 3. (A) L1EV membrane-associated CD9 levels and **(B)** the α-synuclein (α-Syn)/CD9 ratio measured by the droplet assay were similar in neat serum and size exclusion chromatography F3+F4. n = 3 individuals with Parkinson's disease. Data are represented as mean ± SD.

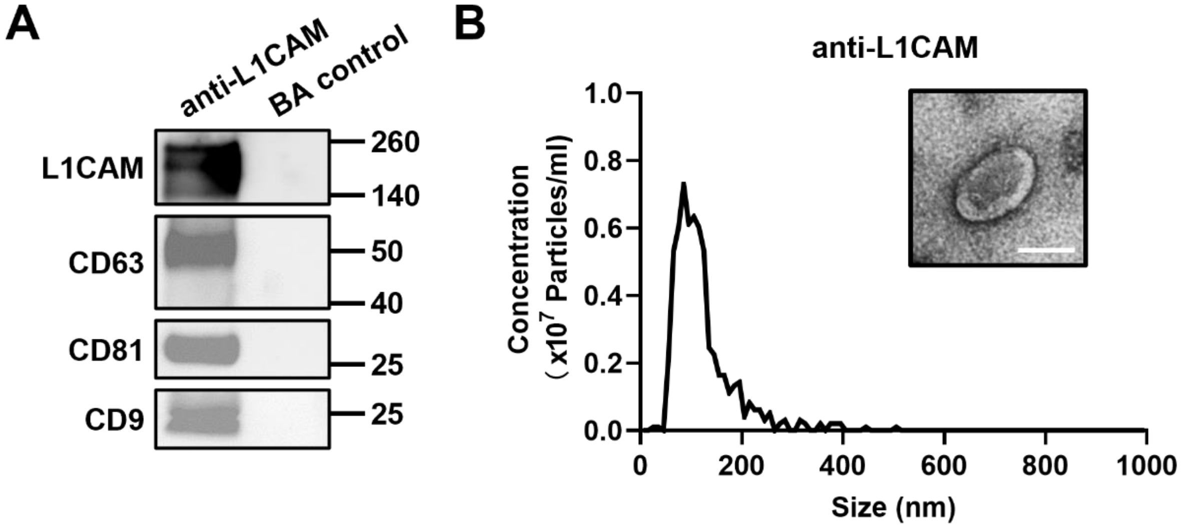

Figure S8. Confirmation of EV isolation from conditioned media. Related to Figure 4. (**A**) Immunocapture using anti-L1CAM from conditioned media of SH-SY5Y cells expressing WT α-synuclein followed by immunoblotting, with beads alone (BA) used as a control. (**B**) Size distribution of eluted EVs after anti-L1CAM immunocapture was confirmed by NTA and the typical cap-shaped appearance was demonstrated by TEM. Scale bar, 100 nm.

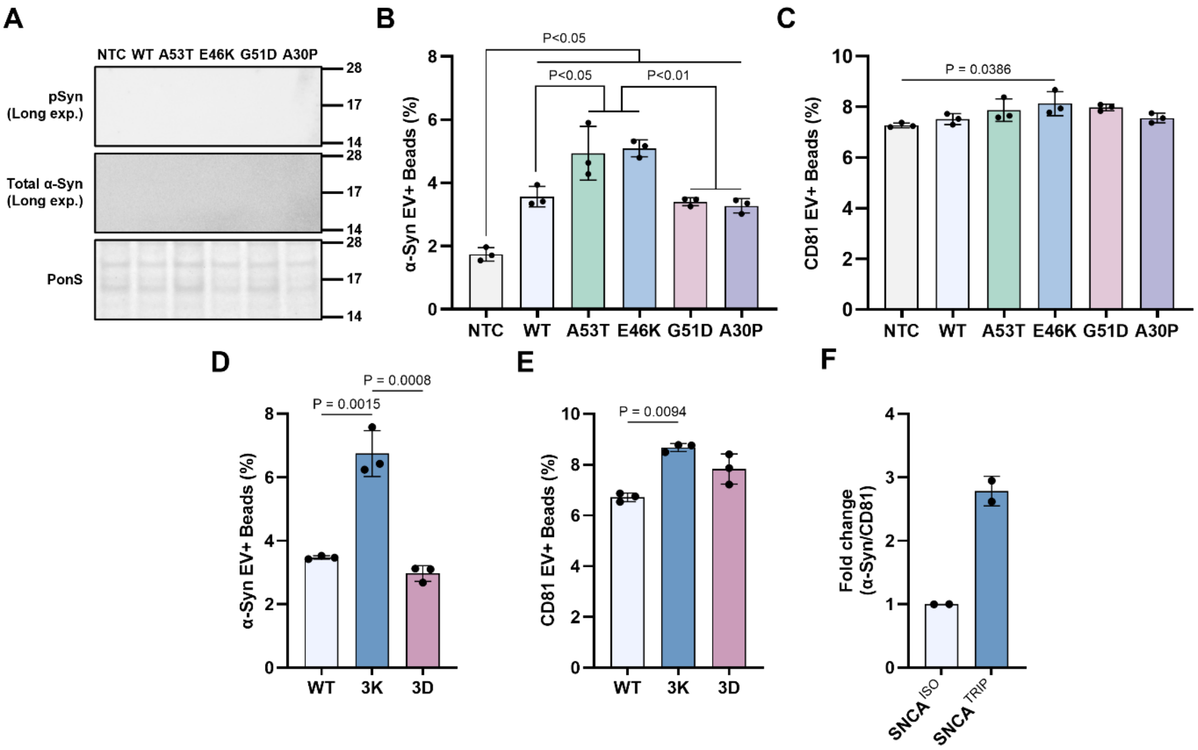

Figure S9. L1EV membrane-associated α-synuclein increases under pathological conditions. Related to Figure 4. (A) pSyn or total α-synuclein (α-Syn) were not detected in RIPA-insoluble 100,000g fraction of non-transduced control SH-SY5Y cells (NTC) and cells expressing α-Syn WT, A53T, E46K, G51D, and A30P. Quantification by the droplet-based assay of **(B)** α-Syn EV positive beads and **(C)** CD81 EV positive beads following anti-L1CAM immunocapture from conditioned media (CM) of NTC and SH-S5Y5 cells expressing α-Syn WT, A53T, E46K, G51D, and A30P; n = 3 independent experiments. **(D)** Quantification by the droplet-based assay of α-Syn and **(E)** CD81 on L1EVs immunocaptured from CM of SH-SY5Y cells expressing α-Syn WT, 3D, and 3K; n = 3 independent experiments. **(F)** The α-Syn to CD81 ratio in the SEC F3+F4 from CM of iPSC dopaminergic neuronal cultures was increased in $SNCA^{TRIP}$ neurons compared to $SCNA^{ISO}$ control as measured by MSD electrochemiluminescence; n = 2 independent differentiations. Data are represented as mean ± SD. Statistical significance was determined by one-way ANOVA (panel **B, C, D,** and **E**).

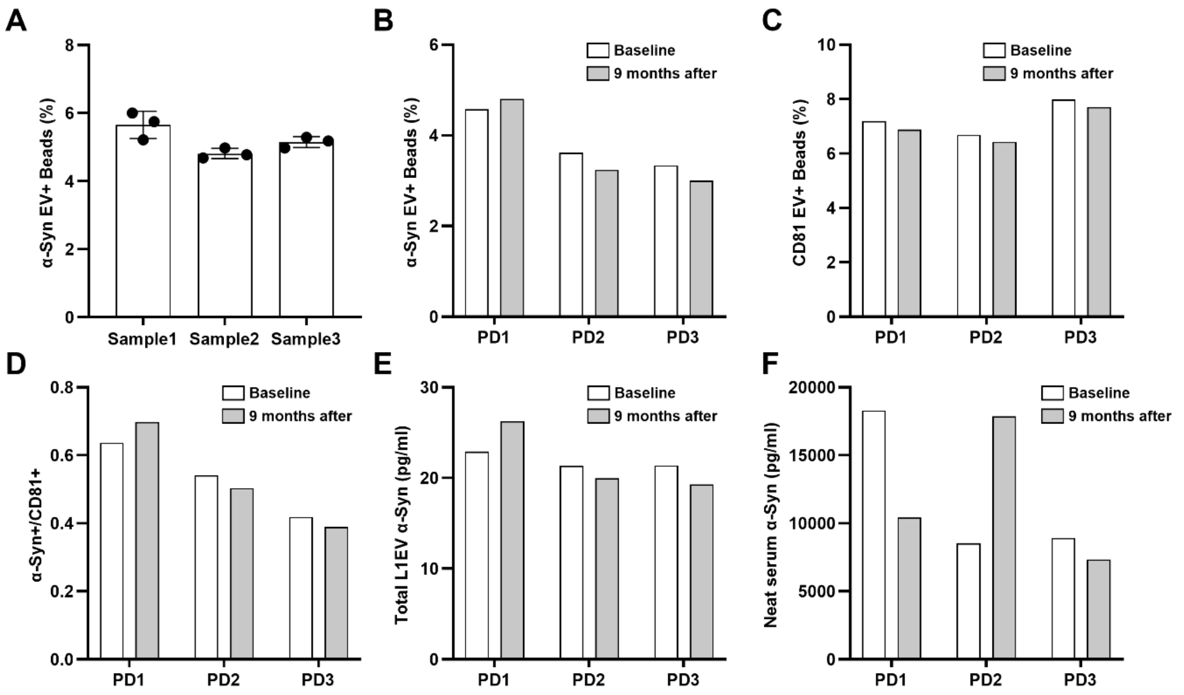

Figure S10. Reproducibility of the assay in detecting L1EV membrane-associated α-synuclein. Related to Figure 5. (**A**) L1EV membrane-associated α-Syn was measured in triplicates from the same sample by the droplet assay, demonstrating the high reproducibility of the assay (n = 3 individuals tested). (**B**) Membrane-associated α-Syn or (**C**) CD81 on serum L1EVs from the same patient, taken 9 months apart, as measured by the droplet assay in three different individuals (PD1, PD2, PD3). (**D**) L1EV membrane-associated α-Syn/CD81 ratio detected by the droplet assay and (**E**) total L1EV α-Syn levels measured by the MSD electrochemiluminescence were stable between serum samples collected 9 months apart, despite variability in (**F**) Soluble/free α-Syn in neat serum measured by the MSD electrochemiluminescence. Data are represented as mean ± SD.

	CD81	α-Syn (A17183A)	α-Syn (LB509)
LOD (L1EV/μl)	66.6	78.9	103.2
LOQ (L1EV/μl)	111.6	131.4	175.9
Dynamic Range (L1EV/μl)	66.6 to 8306.7	78.9 to 4328.0	103.2 to 5758.7
Estimated minimal volume based on LOD (μl)	0.8	2.6	2.4

Table S1. Performance of the assay in detecting serum EV membrane-associated α-Syn and CD81 following anti-L1CAM immunocapture. Related to Figure 3. Abbreviations: α-Syn=α-Synuclein, LOD=limit of detection, LOQ=limit of quantification, L1EV=L1CAM+ EV

ID	Diagnosis	SNCA genotype	Sex	Biopsy age (years)	Original ID	Reprogramming method	iPSC clone characterisation	GEO
SNCA^{TRIP}	PD	Triplication/ WT	female	55	ND34 391G	Cytotune1	Heman-Ackah et al., 2017 doi:10.1093/hmg/ddx331	GSE89886
SNCA^{ISO}	PD	Isogenic WT/WT	female	55	Clone 1-13	Cytotune1	Heman-Ackah et al., 2017 doi:10.1093/hmg/ddx331	GSE89886

Table S2. iPSC clones used in this study. Related to STAR Methods. Abbreviations: PD=Parkinson's disease
